# AGPAT1 is a novel Chikungunya virus receptor on human cells

**DOI:** 10.1128/jvi.01733-25

**Published:** 2026-02-17

**Authors:** Brohmomoy Basu, Debapriyo Sarmadhikari, Anshula Sharma, Shailendra Asthana, Manjula Kalia, Sudhanshu Vrati

**Affiliations:** 1 Regional Centre for Biotechnology 214253, Faridabad, India; 2 Translational Health Science and Technology Institute 145787, Faridabad, India; University of Michigan Medical School, Ann Arbor, Michigan, USA

**Keywords:** virus receptor, RNA virus, alphavirus, Huh7, HAP1, ERMS

## Abstract

Chikungunya virus (CHIKV) is a medically important alphavirus whose host receptor is not fully understood. We identified AGPAT1 from the human Huh7 cell plasma membrane binding with CHIKV particles *in vitro*. The CHIKV binding with AGPAT1 was demonstrated on Huh7 cell plasma membrane by confocal microscopy. The AGPAT1 antibody inhibited CHIKV binding to cells, reducing the virus uptake in Huh7, ERMS, HAP1, and the primary mouse fibroblast cells. CHIKV binding, uptake, and replication were significantly reduced in the AGPAT1 knockout HAP1 cells, and the ectopic expression of AGPAT1 rescued the reduced virus binding, uptake, and replication. AGPAT1, together with CHIKV E2, was found to localize in the early endosomes, early during the virus infection. AGPAT1 interacted with the E1 surface on the E1–E2 dimer of CHIKV envelope proteins *in silico*. The E1-AGPAT1 interacting amino acid residues identified in the computational studies were experimentally verified. AGPAT1 was also shown to have a role in the binding and uptake of another alphavirus, Ross River virus. These data demonstrate an important role for AGPAT1 as a novel host receptor involved in CHIKV binding and uptake in human cells.

**IMPORTANCE:**

Chikungunya virus (CHIKV) is a medically important alphavirus. Identifying its receptor on human cells will aid in the development of novel antivirals. We have identified AGPAT1 as a novel CHIKV receptor on several human cells. We provide data showing the interaction of AGPAT1 with the E1 protein of CHIKV E1-E2 dimer present on the virion surface and identify the amino acid residues involved in the interaction. AGPAT1 was also shown to have a role in the binding and uptake of Ross River virus, another alphavirus. AGPAT1 is a protein involved in lipid metabolism and has not been reported to have a direct role in any virus infection or as a virus receptor. Thus, this work identifies AGPAT1 as a novel receptor for CHIKV on human cells and demonstrates that AGPAT1 has implications for understanding the virus pathogenesis and designing the receptor-blocking CHIKV antivirals.

## INTRODUCTION

Chikungunya virus (CHIKV) is an alphavirus transmitted mainly by *Aedes aegypti* and *Aedes albopictus* mosquitoes (1). CHIKV causes high fever, maculopapular rashes, debilitating pain, and polyarthralgia. A major outbreak of chikungunya fever occurred in India in 2006, where 1.25 million cases were reported (2). In the last 15 years, the virus has spread to over 110 countries in Asia, Europe, South and Central America, and Africa (3). Although CHIKV is not a deadly virus, it causes severe, long-lasting indisposition in a large population each year and is thus considered a priority pathogen. A live, attenuated CHIKV vaccine was approved recently by the U.S. Food and Drug Administration (4, 5). However, no CHIKV-specific antivirals are available.

CHIKV has a single-stranded, positive-sense, ~12 kb long RNA genome encoding four non-structural and five structural proteins (C, E3, E2, 6K, and E1). The dimers of the E1 and E2 proteins, ultimately forming trimers, are present as spikes on the virion surface (6). The E1 protein, a 47-kDa transmembrane protein, is involved in the fusion step during infection. The 50-kDa transmembrane E2 protein induces neutralizing antibodies (7, 8). Both E1 and E2 are involved in receptor binding (9–11).

CHIKV infects different animal hosts such as mosquitoes (12), humans (13), monkeys (14), rodents (15), bats (16), birds (17), and livestock animals (18). Besides, the virus can infect a variety of cell types, such as fibroblasts (19), myocytes (20), epithelial cells (19), macrophages (21), and dendritic cells (22). The variety of CHIKV-susceptible host species and cell types suggests that the virus might use diverse cell surface molecules as an attachment factor or receptor.

Several attachment factors have been identified for alphaviruses. These include glycosaminoglycans (GAGs) such as heparan sulfate (23) and chondroitin sulfate (24). The 181/25 CHIKV isolate needed heparan sulfate for its infectivity, whereas the LR2006 OPY1 CHIKV strain showed dependence upon heparan sulfate proteoglycans (HSPGs) or other GAGs for infectivity (22). While the cell-surface GAGs promote viral entry, they are not essential for CHIKV infection. CHIKV can enter GAG-deficient cells, suggesting GAG-independent entry pathways exist (25). Thus, the attachment factor, GAGs, mainly enhances the virus infection.

Studies have identified several potential cell surface receptors facilitating CHIKV entry into the host cell (26). Thus, the Matrix Remodeling Associated 8 (MXRA8) protein serves as a receptor for CHIKV and other arthritogenic alphaviruses in mouse and human cells (27). Another protein, Prohibitin, was shown to be involved in the uptake of CHIKV in the human microglial CHME-5 cells (28). In 293T cells, the CD147 protein complex acted as a possible entry factor for CHIKV and other alphaviruses (29). Besides, TIM-1 and DC-SIGN have been shown as the CHIKV attachment factors on mammalian cells (30, 31).

These studies illustrate the complex interactions between CHIKV and the host cell surface proteins, enabling viral entry and infection. None of the proteins described above serves as the exclusive receptor for CHIKV entry into the host cell, and more CHIKV receptor proteins remain to be discovered. Identification of CHIKV receptors in mammalian cells will help develop the virus entry inhibitors as novel antivirals. Here, we describe the 1-acyl-sn-glycerol-3-phosphate acyltransferase alpha (AGPAT1) protein that acts as a novel CHIKV receptor on Huh7, HAP1, and embryonal rhabdomyosarcoma (ERMS) human cells.

## RESULTS

### CHIKV binds AGPAT1 protein from the Huh7 plasma membrane *in vitro*

The Huh7 plasma membrane proteins’ binding with purified CHIKV was carried out *in vitro,* and the interacting host proteins were identified by mass spectrometry. From the six independent experiments, the CHIKV-binding proteins identified on two or more occasions are shown in Fig. S1. However, Junction plakoglobin (JUP) and Filaggrin (FLG) were seen in three, Annexin 2 (ANXA2) and Hornerin (HRNR) in four, and AGPAT1 was recorded in five experiments.

### AGPAT1 interacts with CHIKV on the Huh7 plasma membrane *in cellulo*

**Fig 1:**
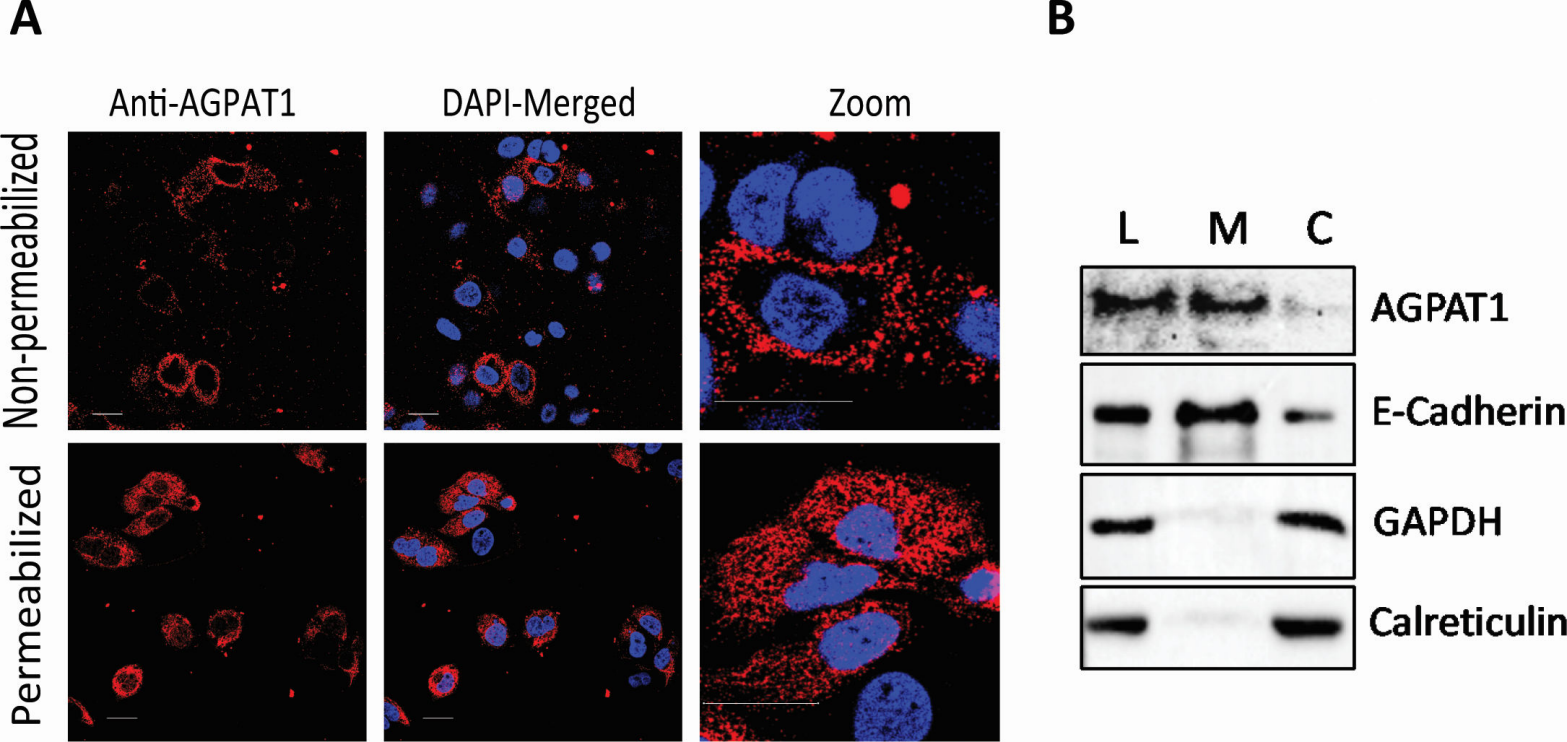
AGPAT1 is present on the plasma membrane of Huh7 cells. (**A**) Huh7 cells were permeabilized with 0.3% Tween-20 or were not permeabilized. The cells were then incubated with rabbit AGPAT1 antibody, followed by fixing the cells in 2% paraformaldehyde and staining with Alexa Fluor-568 antibody. The nuclei were stained with 4′,6-diamidino-2-phenylindole (DAPI), and the cells were imaged. (**B**) Huh7 cells were grown as monolayers, and the total cell lysate (L) was prepared. The plasma membrane (M) and cytoplasmic (C) fractions of the cells were prepared and western blotted. The presence of AGPAT1 in the membrane fraction is seen. E-cadherin, GAPDH, and calreticulin were used as controls to demonstrate the protein fractionation.

AGPAT1 is reported on the mammalian cell endoplasmic reticulum membrane (32, 33). To function as a CHIKV cellular receptor, it must be located on the plasma membrane. Indeed, AGPAT1 was located on the Huh7 plasma membrane, as revealed by immunofluorescence imaging of the non-permeabilized cells (Fig. 1A). Western blotting of the cytoplasmic and membrane fractions (devoid of the ER membranes) confirmed the presence of AGPAT1 on the Huh7 plasma membrane (Fig. 1B).

**Fig 2:**
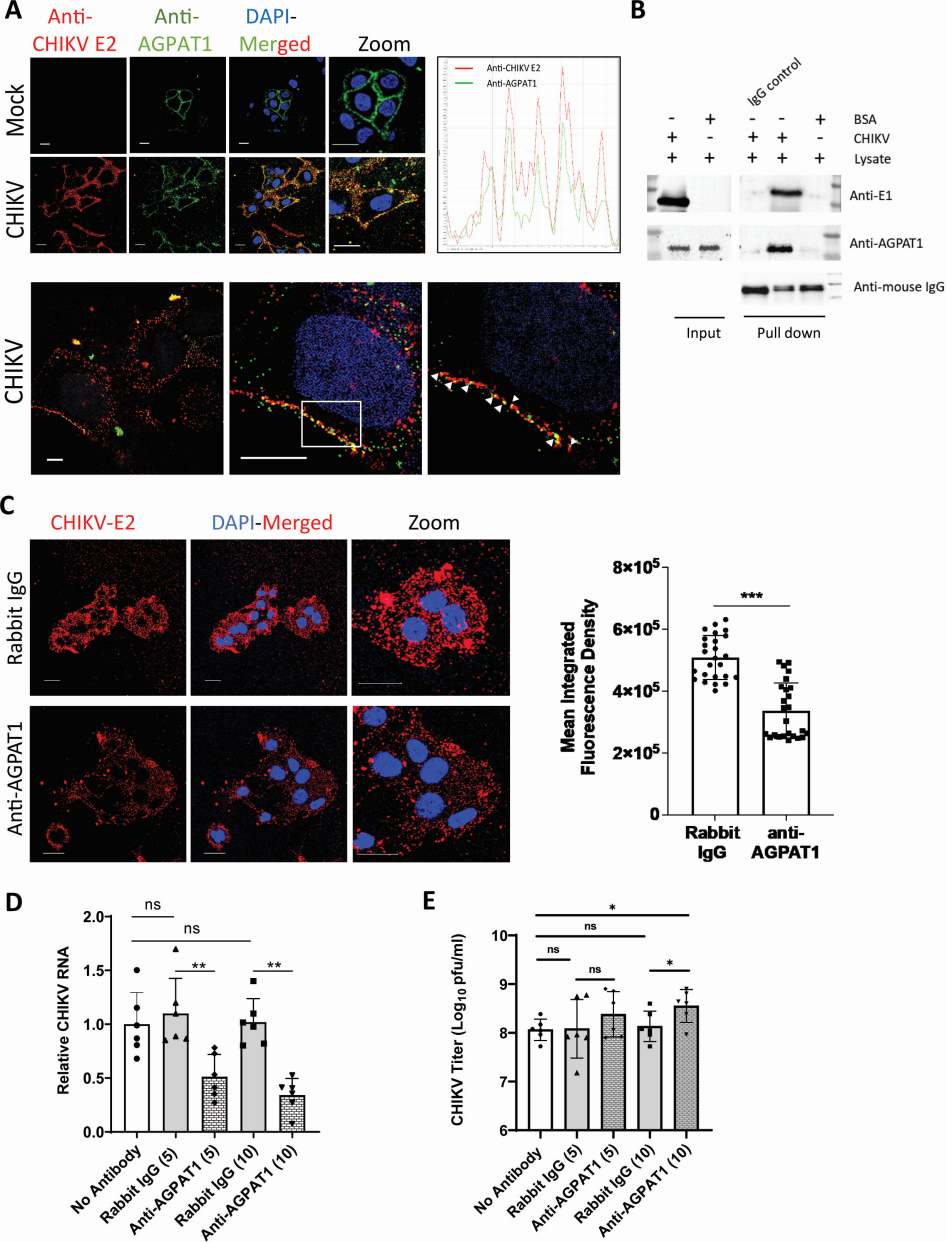
AGPAT1 interacts with CHIKV on the Huh7 plasma membrane. (**A**) Huh7 cells were incubated with CHIKV (multiplicity of infection [MOI] 50) on ice for 45 min, followed by CHIKV anti-E2 mouse monoclonal antibody and anti-AGPAT1 rabbit polyclonal antibody for another 30 min. The cells were washed with ice-cold phoshate-buffered saline (PBS) and incubated with anti-mouse Alexa Fluor-647 and anti-rabbit Alexa Fluor-488 antibodies for 30 min. The nuclei were stained with DAPI, and the cells were imaged as shown in the top left panel. The PCC was determined for the co-localization of Alexa Fluor-488 (AGPAT1) and Alexa Fluor-647 (CHIKV) on the plasma membrane. The top right panel shows the line plot showing the co-localization of the red (CHIKV) and green (AGPAT1) fluorescence signals. The bottom panel shows the colocalization of CHIKV and AGPAT1 observed by the super-resolution structured illumination microscopy (SIM) under the Zeiss Elyra PSI microscope (scale bar = 10 µm). The white arrows show the co-localized puncta under 100× magnification and 10× zoom. (**B**) The Huh7 cell lysate was incubated with the purified CHIKV or BSA (negative control). The immunoprecipitation was carried out using the CHIKV anti-E1 antibody or the isotype control IgG. The input cell lysate and the pull-down were western blotted with AGPAT1 or CHIKV E1 antibodies. (**C**) Huh7 cells were incubated on ice with rabbit anti-AGPAT1 polyclonal antibody or rabbit IgG (isotype control) for 30 min at 10 µg/mL concentration, followed by incubation with CHIKV (MOI 50) on ice for 30 min. The cells were then incubated with CHIKV anti-E2 mouse monoclonal antibody on ice for 30 min and washed with ice-cold PBS. The cells were stained with Alexa Fluor-647 anti-mouse antibody for 30 min. The nuclei were stained with DAPI, and the cells were imaged. The MIFD (25 regions of interest [ROI]) is shown as mean±SD in the right panel. The experiment was done in triplicate. (**D**) Huh7 cells were incubated with 5 or 10 μg/mL concentration (indicated in the brackets) of rabbit anti-AGPAT1 antibody or rabbit IgG (isotype control) on ice for 30 min, or without antibody, and then incubated with CHIKV (MOI 1) on ice for 1 h. The cells were then washed with ice-cold PBS and incubated for 3 h at 37°C. The total RNA from the cells was isolated, and the CHIKV RNA level was determined by qRT-PCR. The CHIKV RNA level in the no-antibody control was taken as 1. The data from three biological replicates and two technical replicates are shown. (**E**) Huh7 cells were incubated with 5 or 10 µg/mL concentration (indicated in the brackets) of rabbit anti-AGPAT1 antibody or rabbit IgG (isotype control) on ice for 30 min, or without antibody, and then incubated with the inoculum containing CHIKV (MOI 1) on ice. The inoculum was removed 1 h later, and the CHIKV titer was determined. The data from three biological replicates and two technical replicates are shown. *P < 0.05, **P < 0.01, ***P < 0.001, or ns = not significant.

Immunofluorescence-based confocal microscopy showed colocalization (PCC 0.62) of CHIKV virions and AGPAT1 on the plasma membrane (Fig. 2A). The super-resolution structured illumination microscopy corroborated this finding (Fig. 2A, lower panel). This was further confirmed by immunoprecipitation of AGPAT1 from the Huh7 plasma membrane fraction incubated with purified CHIKV (Fig. 2B).

CHIKV binding to Huh7 cells was studied by measuring the mean integrated fluorescence density (MIFD) on cells incubated with CHIKV, followed by immunofluorescence staining and confocal microscopy. The MIFD was reduced (*P* < 0.001) by 34% on cells incubated with CHIKV in the presence of AGPAT1 antibody when compared with the control (Fig. 2C), reinforcing the finding that CHIKV binds to AGPAT1 on Huh7 plasma membrane. Corroborating this, CHIKV infection of Huh7 cells was reduced (*P* < 0.01) by 53% and 68% in the presence of 5 µg/mL and 10 µg/mL AGPAT1 antibody in a dose-dependent manner, respectively (Fig. 2D). Here, the rabbit IgG (isotype control) did not affect the virus infection. In this experiment, CHIKV RNA and the viral titers measured at 6 h post-infection were significantly reduced in the presence of AGPAT1 antibody (Fig. S2).

In another experiment, CHIKV binding to Huh7 cells was allowed in the presence or absence of the AGPAT1 antibody, and the inoculum was titrated 1 h later for the unbound virus (Fig. 2E). More virus remained unbound (*P* < 0.05) in the inoculum during CHIKV binding to the cells in the presence of the AGPAT1 antibody, suggesting that AGPAT1 antibody inhibited CHIKV binding to Huh7 cells.

### CHIKV uptake in Huh7 cells is related to AGPAT1 protein levels

**Fig 3:**
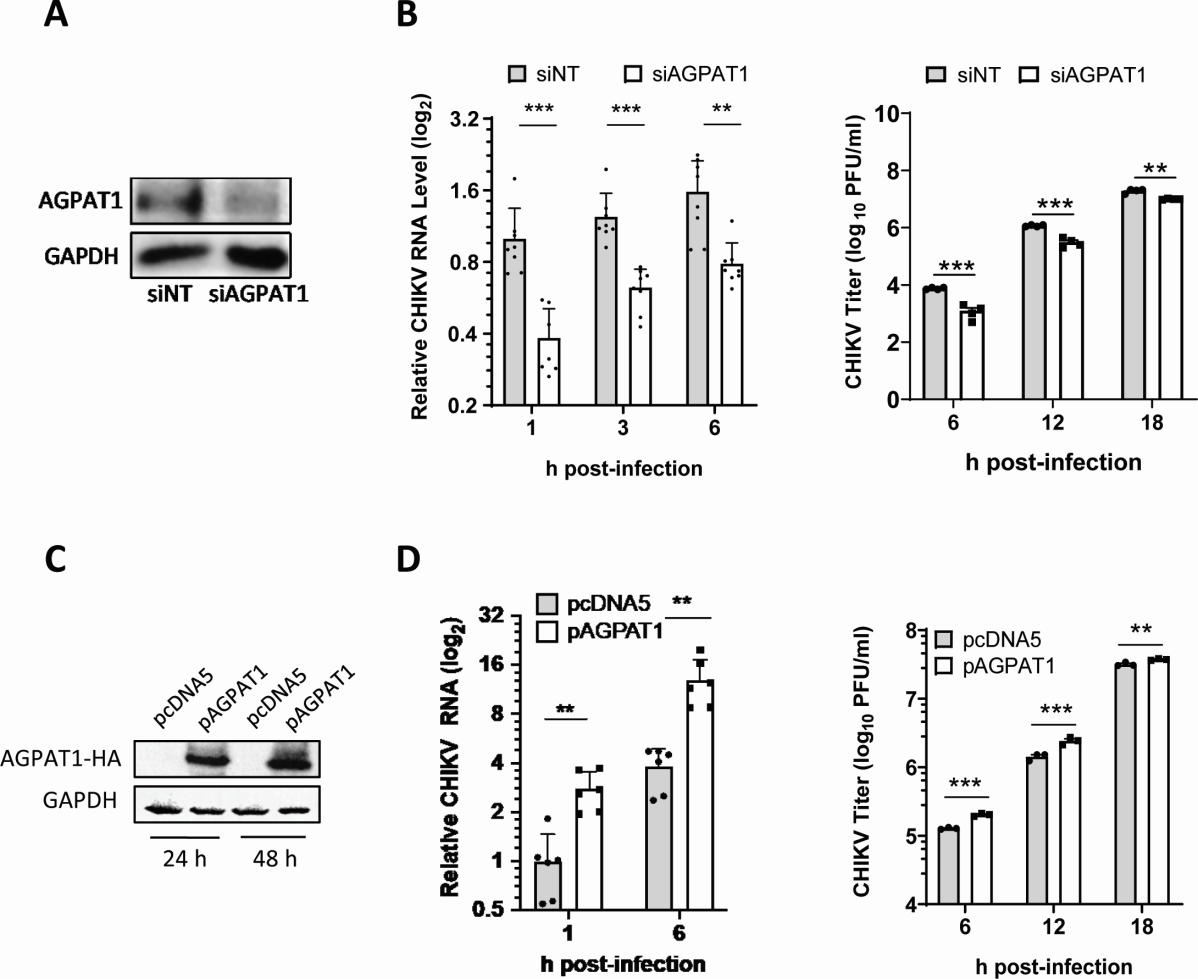
CHIKV uptake in Huh7 cells is related to the AGPAT1 protein levels. (**A**) Huh7 cells were transfected with siRNA to AGPAT1 (siAGPAT1) or a control non-targeting siRNA (siNT) and incubated for 48 h at 37°C. Western blotting showed a ~60% reduction in the AGPAT1 levels in the siAGPAT1-treated cells. (**B**) The siRNA-treated cells were incubated 48 h post-transfection (pt) with CHIKV (MOI 1) for 1 h on ice for the virus uptake assay. The relative CHIKV RNA levels are shown (left panel), where the CHIKV RNA level in the siNT-transfected cells at 1h post-infection (pi) was taken as 1. The viral titers determined by plaque assay are shown in the right panel. The data from four biological replicates and two technical replicates are shown. (**C**) Huh7 cells were transfected with the pAGPAT1 plasmid expressing the HA-tagged AGPAT1 or the empty vector pcDNA5. The cells were harvested at 24 and 48 h later. The cell lysates were western blotted with monoclonal anti-HA antibody to detect the expression of the HA-tagged AGPAT1. GAPDH was used as the loading control. (**D**) Huh7 cells were transfected with pAGPAT1-HA or pcDNA5, and 48 h later, they were incubated with CHIKV (MOI 5) for the virus binding assay. The relative CHIKV RNA levels are presented (left panel), where the CHIKV RNA level at 1 h pi was taken as 1. The right panel has the virus titers. The data from three biological replicates and two technical replicates are shown. **P < 0.01, ***P < 0.001.

The siRNA-mediated knockdown of AGPAT1 was used to study its role in CHIKV uptake (Fig. 3A). The siRNA treatment did not affect cell viability. The CHIKV RNA levels were lowered (*P* < 0.001) by 62%, 50%, and 48% in the siAGPAT1-treated CHIKV-infected cells compared to the control at 1, 3, and 6 h pi, respectively, showing a 62% reduced virus uptake (Fig. 3B). The viral titers were 83%, 73%, and 48% lower (*P* < 0.001) in the siAGPAT1-treated cells than in the control at 6, 12, and 18 h pi, respectively. Furthermore, CHIKV uptake was studied in Huh7 cells ectopically expressing the HA-tagged protein AGPAT1-HA (Fig. 3C). A significantly enhanced (*P* < 0.01) level of viral RNA (175% and 240% higher at 1 and 6 h pi, respectively) was seen in AGPAT1-overexpressing CHIKV-infected cells compared to the empty vector-transfected control cells, showing a 175% enhanced virus uptake. Similarly, higher titers (*P* < 0.001) of CHIKV (175%, 240%, and 120% higher at 6, 12, and 18 h pi, respectively) were seen in AGPAT1-overexpressing CHIKV-infected cells (Fig. 3D).

### CHIKV binding and uptake are reduced in the AGPAT1 knockout (KO) HAP1 cells

**Fig 4:**
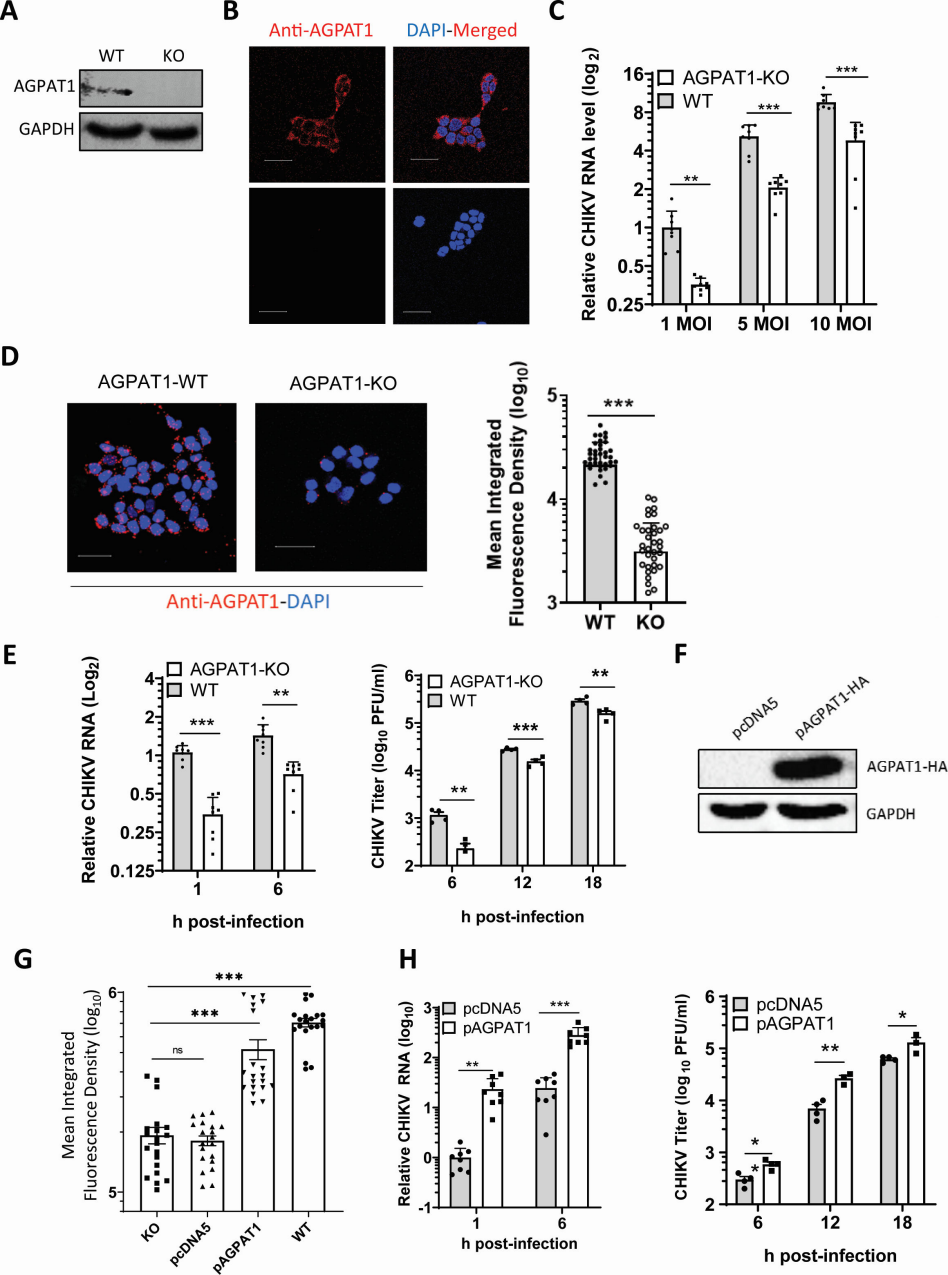
CHIKV binding and uptake in AGPAT1 knockout HAP1 cells. (**A**) The wild-type (WT) and AGPAT1 knockout (KO) HAP1 cell lysates were western-blotted with anti-AGPAT1 antibody to establish the absence of AGPAT1 in the KO cells. GAPDH was used as the loading control. (**B**) The HAP1 KO and WT cells were incubated with rabbit anti-AGPAT1 antibody and fixed by 2% paraformaldehyde, followed by incubation with anti-rabbit Alexa Fluor-568 antibody. The nuclei were stained with DAPI, and the cells were imaged. (**C**) The KO and WT HAP1 cells were incubated with CHIKV at the indicated MOI for the virus binding assay. The relative CHIKV RNA levels indicating the virus binding are presented, where the CHIKV RNA level at 1 MOI in the WT cells was taken as 1. The data from four biological replicates and two technical replicates are shown. (**D**) CHIKV binding to AGPAT1 KO and WT HAP1 cells was studied by incubating the cells with CHIKV (MOI 25), followed by staining with CHIKV anti-E2 mouse antibody and Alexa Fluor-647 anti-mouse antibody, and imaging for MIFD (45 ROI) determination. The MIFD data are from three biological replicates. (**E**) The WT and AGPAT1 KO HAP1 cells were incubated with CHIKV (MOI 0.1) and processed for the virus uptake assay. The relative CHIKV RNA levels are presented (left panel), where the CHIKV RNA level at 1 h pi in the WT cells was taken as 1. The right panel has the virus titers. The data from four biological replicates and two technical replicates are shown. (**F**) The AGPAT1-KO HAP1 cells were transfected with pAGPAT1-HA or pcDNA5 and incubated for 48 h at 37°C. The western blotting showed the ectopic expression of the AGPAT1-HA protein in the transfected cells. (**G**) The HAP1 AGPAT1 KO cells transfected with pAGPAT1-HA or pcDNA5 as above were fixed and incubated with CHIKV (MOI 25) on ice for 30 min. This was followed by staining the cells with CHIKV anti-E2 mouse antibody and microscopy for MIFD (20 ROI) determination. The MIFD data from three biological replicates are shown. (**H**) The AGPAT1 KO HAP1 cells were transfected with the plasmid pAGPAT1-HA or pcDNA5, and 48 h later, they were incubated with CHIKV (MOI 5) for 1 h on ice for the virus uptake assay. The relative CHIKV RNA levels are presented (left panel), where the CHIKV RNA level at 1 h pi in the pcDNA5-transfected cells was taken as 1. The right panel has the virus titers. The data from four biological replicates and two technical replicates are shown. *P < 0.05, **P < 0.01, ***P < 0.001, or ns = not significant.

HAP1 is a near-haploid human cell line. The AGPAT1 KO HAP1 cell line was generated and validated for the absence of AGPAT1 by western blotting (Fig. 4A). Compared to the WT cells, no apparent differences in the health or growth rate of AGPAT1 KO cells were noted. Notably, immunofluorescence staining for AGPAT1 was seen on the plasma membrane of the wild-type (WT) HAP1 cells, whereas it was absent on the KO cells (Fig. 4B).

Compared to the WT HAP1 cells, the KO cells showed 64%, 60%, and 50% reduction (*P* < 0.001) in CHIKV binding at MOI 1, 5, and 10, respectively (Fig. 4C). The confocal microscopy (Fig. 4D) showed that CHIKV binding, measured as the MIFD, was ~85% lower (*P* < 0.001) in the KO cells than in the WT cells. CHIKV RNA levels in the KO cells showed 71% reduction both at 1 and 6 h pi compared to the WT cells, showing a 71% reduced virus uptake (Fig. 4E). Notably, the virus replication, measured as the extracellular virus titers, was reduced (*P* < 0.001) by 80%, 44%, and 45% at 6, 12, and 18 h pi, respectively, in the KO cells compared to the WT cells (Fig. 4E).

To see if the reduced CHIKV binding, uptake, and replication in the KO HAP1 cells could be rescued by the ectopically expressed AGPAT1, the cells were transfected with the pAGPAT1-HA expression vector or with the control empty vector pcDNA5. The western blotting established the expression of AGPAT1-HA in the transfected cells (Fig. 4F). The CHIKV binding, determined by MIFD, was 200% higher (*P* < 0.001) in the KO cells ectopically expressing AGPAT1-HA than in the KO cells (Fig. 4G; Fig. S3). In total, 23-fold and 12-fold enhanced (*P* < 0.01) viral RNA levels were seen in the AGPAT1-expressing KO cells than in the control at 1 and 6 h pi, respectively (Fig. 4H), showing a 2,300% enhanced CHIKV uptake. Similarly, CHIKV replication was enhanced (*P* < 0.01) in the AGPAT1-expressing HAP1 KO cells. Thus, CHIKV titers were 100%, 300%, and 100% higher in the rescued cells compared to the control at 6, 12, and 18 h pi (Fig. 4H).

These data show that CHIKV binds AGPAT1 on the HAP1 plasma membrane, and the virus binding, uptake, and replication are significantly reduced in the cells lacking AGPAT1.

### *In silico* analysis shows AGPAT1 interaction with CHIKV E1 protein

**Fig 5:**
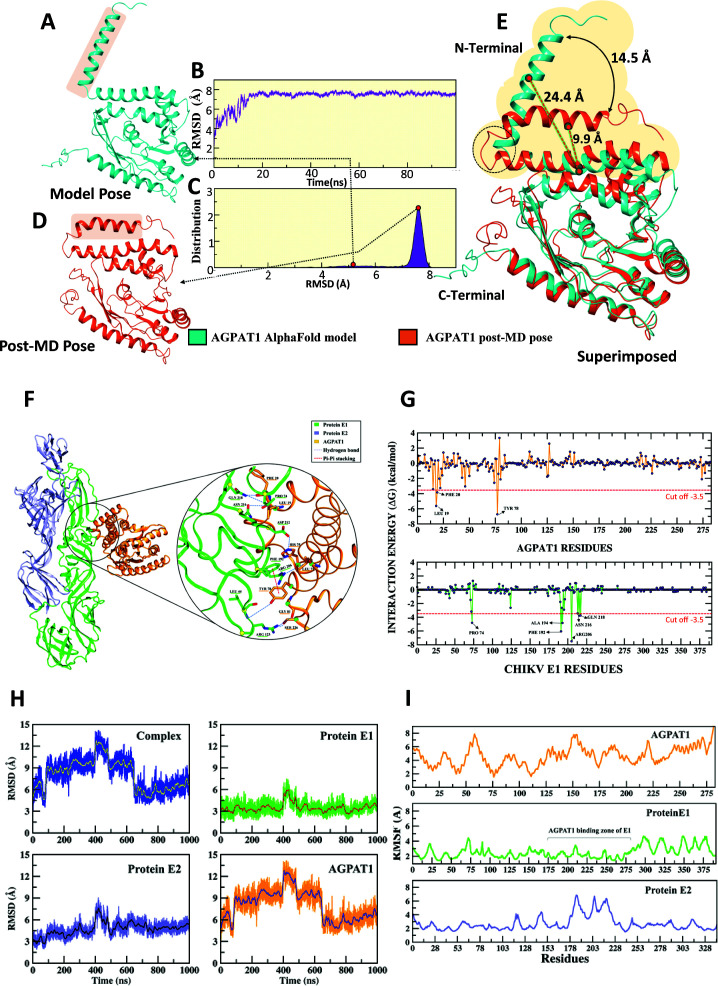
AGPAT1 interaction with the CHIKV E1-E2 dimer *in silico*. (**A**) AGPAT1 structure derived using AlphaFold 3 (model pose). (**B**) Time-dependent evolution of AGPAT1 (model pose) during MD simulation. The structural conformation of the protein over time was studied as RMSD. (**C**) Histogram of RMSD. (**D**) Stable MD pose of AGPAT1. (**E**) Overlay of model *vs*. MD pose. The confirmational change of the N-terminal is depicted, and the distance between the two poses is presented. The dotted circle indicates the hinge-like motion because of the loop that exists between two helices. The model and MD poses are rendered in cartoon and colored in cyan and orange, respectively. The major conformational change region is highlighted in panels **A**, **D**, and **E**. (**F**) The pictorial representation of the CHIKV E1-E2 dimer interaction with the AGPAT1 protein is shown. Interactions in the form of hydrogen bonds and Pi-Pi interactions are depicted. (**G**) The amino acid residues at the complex interface site were analyzed by the molecular mechanics generalized born surface area (MM-GBSA) approach. Per-residue energy breakdown analysis was used to determine the energy contribution of individual amino acids to identify critical residues at the interface and reveal primary residue interactions within the complex by decomposing binding free energy (kcal/mol). Residues with higher interaction energies (cutoff => −3.5 kcal/mol) are identified; these are predicted to be involved in strong binding. (**H**) The structural conformation of the complex over time was studied as RMSD, calculated using the backbone atoms of the whole complex (E1–E2 dimer with AGPAT1). Individual backbone RMSD of each protein present in the complex is also depicted. (**I**) The atomic level fluctuation for the individual protein in the complex was studied as root mean square fluctuation (RMSF). Significantly less fluctuation in the AGPAT1 binding zone of the E1 protein is highlighted.

CHIKV has E1 and E2 proteins on the virion surface that could interact with AGPAT1, together or individually. The CHIKV E1–E2 dimer structure from the protein data bank (PDB-ID: 3N42) was used. The AGPAT1 structure was obtained using AlphaFold 3 (Fig. 5A). The AlphaFold-predicted AGPAT1 structure was used to run a 100 ns all-atom MD simulation to assess the stability of the apo structure. The root mean square deviation (RMSD) value of the trajectory revealed that the structure underwent a rapid rise in RMSD during the initial stages of the simulation, followed by a gradual increase that plateaued at ~7–8 Å after around 20 ns, indicating a significant conformational rearrangement relative to the starting AlphaFold model (Fig. 5B). Furthermore, the RMSD distribution showed a significant peak at ~8.0 Å (Fig. 5C), which indicated that the apo structure attained a stable conformational ensemble. Superimposition of the initial AlphaFold model (Fig. 5A) and the post-molecular dynamics (MD) structure (Fig. 5D) highlighted significant movements within the N-terminal region of AGPAT1 structure (Fig. 5E). The N-terminal region exhibited an outward displacement of approximately 24.4 Å from its adjacent helical region in the AlphaFold model; however, after MD simulation, it reduced to 9.9 Å, indicating a significant shift of 14.5 Å, which helps the protein to obtain a stable conformation. Such rearrangements likely reflect the intrinsic flexibility of the AGPAT1 protein required for substrate accommodation.

To obtain the most likely complex, the interaction of the CHIKV E1 and E2 dimer with AGPAT1 stable pose obtained from apo MD (Fig. 5D) was studied *in silico*. A total of 100 protein-protein docking poses were generated using HDOCK. To rule out any HDOCK bias, complexes using AlphaFold 3 were also generated. To identify the most likely complexes, the top five HDOCK poses showing higher dock score and top five AlphaFold 3 poses showing higher confidence score (Fig. S4; Table S2) were analyzed using MM-GBSA for a comparative binding free energy (Table S3). Based on the higher MM-GBSA score, HDOCK pose 1 and pose 2 were selected for further analysis; however, none of the AlphaFold 3 poses were selected due to their lower MM-GBSA scores. The two HDOCK poses were used for detailed residue-wise interaction analysis between E1 and E2 dimers with AGPAT1 to map the critical residues involved in establishing the stable complex. The per-residue decomposition was also carried out to identify the major contributing residues in terms of energetics. We observed that pose 2 of AGPAT1 interacted with the domain II of the E1 protein (Fig. 5F and G), which was previously reported to interact with the MXRA8 protein (9); therefore, pose 2 was preferred. The CHIKV envelope spike comprises a trimer of E1–E2 dimers (34). Therefore, the E1–E2 dimer in complex with AGPAT1 was superimposed onto a single spike of the CHIKV envelope (Fig. S5). Superimposition of the pose 2 complex with the trimeric spike revealed that AGPAT1 remained positioned near the cell membrane-facing side of the trimer, whereas adequate spatial clearance was retained to accommodate AGPAT1. The predicted interaction interface was concentrated in the plasma membrane-proximal regions of E1, which remained accessible in the trimeric context.

Based on the support from the previous experimental finding of MXRA8 interaction with CHIKV envelope (9), and due to a considerably higher MM-GBSA score (Table S3), pose 2 was studied further for the complex conformational stability using the MD simulations.

### MD simulation of the AGPAT1-CHIKV E1–E2 dimer complex

The MD simulation was carried out to understand the stable state of the docked complex with respect to time. Post-MD analysis techniques, like RMSD and RMSF, were used for the quantitative and qualitative measurements of the MD trajectories. The RMSD study indicated that the complex achieved its stable state (plateau) in a 1,000 ns MD simulation run. The dynamic behavior of the complex system during the MD simulations revealed that although the structure experienced initial fluctuations, it stabilized after ~600 ns (Fig. 5H). This suggested that in the later stages of the simulation, protein-protein interactions contributed to reducing the structural deviations. Furthermore, a comparison between the initial (0 ns) and final (1,000 ns) structures of the complex showed a minimal fluctuation (RMSD ~2.8 Å), indicating the structural robustness and stability of the docked complex.

The individual RMSD profiles of the E1–E2 dimer and AGPAT1 were also evaluated (Fig. 5H). The results indicated that the E1–E2 dimer maintained a stable conformation from the beginning, whereas AGPAT1 showed some initial fluctuations but stabilized around ~600 ns. This suggested that the observed fluctuations in the AGPAT1-CHIKV E1–E2 complex were primarily due to AGPAT1.

The MM-GBSA analysis was carried out on the stable trajectory to embrace the outcomes of the MD simulations. The results indicated that AGPAT1 had a high binding affinity of −85.81 kcal/mol toward the CHIKV E1-E2 dimer. This high binding affinity indicates the robustness of the complex, which kept the complex stable during the MD simulation.

Protein flexibility is an intrinsic characteristic that can influence various biological processes. The RMSF analysis (Fig. 5I) showed that the E1–E2 dimer interaction with AGPAT1 reduces the protein flexibility at the binding regions (marked in Fig. 5I). Here, AGPAT1 primarily interacted with the E1 component of the E1–E2 dimer to gain high stability with an average Cα RMSF value of ~2.0 Å, showing a rigid conformation with minimal fluctuation.

### Interaction fingerprinting identifies the key amino acids in the AGPAT1-CHIKV E1–E2 complex

Stable interactions observed between the CHIKV E1–E2 dimer and the AGPAT1 protein throughout the MD simulation were primarily stabilized by strong hydrogen bonds and a notable π–π stacking. Importantly, the majority of the AGPAT1’s contacts were established with domain II of the E1 protein. A total of eight hydrogen bonds (see below) and one π–π stacking interaction (Phe192@Tyr78) were identified as key stabilizing forces during the simulation (Fig. 5F). Interaction fingerprint analysis of the most stable conformational state (which is close to the average structure of the lowest RMSD) obtained from the MD trajectory revealed the specific residues involved in the complex formation (Fig. 5F). Within the 4.0 Å distance, AGPAT1 residues closely associated with six E1 residues. The *in silico* interaction analysis, followed by the per-residue energy decomposition analysis, identified critical residues involved in the AGPAT1 interaction with the E1–E2 dimer (Fig. 5G). These included the hydrophobic (Leu73), aromatic (Pro74, Phe192, and Tyr214), basic (Arg123 and Arg206), and polar (Asp212, Asn216, and Gln218) amino acids. Among these residues, Arg123, Arg206, Asp212, Asn216, and Gln218 were involved in making stable hydrogen bonds with AGPAT1 residues. The AGPAT1 residues Leu19, Phe20, Ser22, Phe47, Leu71, His75, Tyr78, and Gly81 were in close contact with the E1 protein, contributing to the overall stability of the complex. The key CHIKV E1 and AGPAT1 interactions, which were durable during the MD simulations, more than 30% were identified as Phe192@Tyr78, Arg206@Leu71, Leu44@Tyr78, Gln218@Leu19, Asp212@His75, Gln218@Pro74, and Asn216@Leu19.

### CHIKV E1 interacts with AGPAT1 in Huh7 cells

To validate the *in silico* findings, confocal microscopy-based immunofluorescence colocalization studies were conducted. Colocalization of CHIKV E1 and the endogenous AGPAT1 (PCC 0.63) was observed in CHIKV-infected Huh7 cells (Fig. S6A). Since E1 and E2 may exist as dimers in CHIKV-infected cells, it cannot be reliably inferred that AGPAT1 interacts with CHIKV E1. To address this, Huh7 cells were transfected with the plasmid expressing CHIKV E1 carrying the FLAG tag. Here, we observed the colocalization (PCC 0.69) of the endogenous AGPAT1 with the ectopically expressed CHIKV E1-FLAG protein (Fig. S6B). To corroborate these findings, Huh7 cells were transfected with the plasmids expressing CHIKV E1-FLAG and AGPAT1-HA proteins. These cells showed strong colocalization (PCC 0.79) of the ectopically expressed CHIKV E1-FLAG and AGPAT1-HA proteins (Fig. S6C), whereas CHIKV-E2-FLAG did not colocalize (PCC 0.30) with AGPAT1-HA protein (Fig. S6D). These findings were supported by co-immunoprecipitation of CHIKV E1 and AGPAT1 proteins from Huh7 cells (Fig. S6E and F).

### The mutants of the CHIKV E1 and AGPAT1 proteins show a reduced interaction

**Fig 6:**
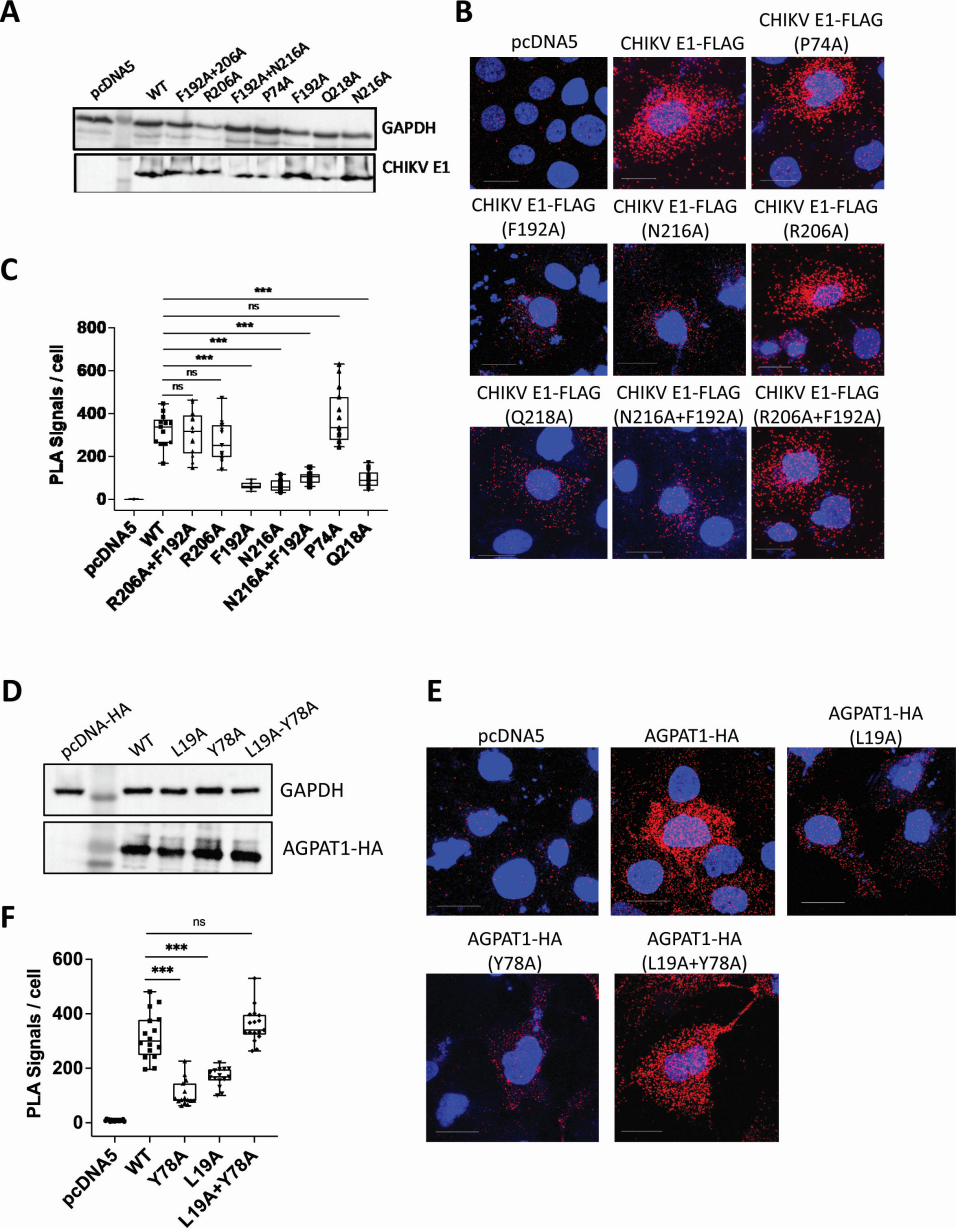
Interaction of the mutants of CHIKV E1 and AGPAT1 proteins. (**A**) Huh7 cells were co-transfected with pAGPAT1-HA and the plasmids expressing the CHIKV E1-FLAG protein (WT) or its mutants, or the empty vector pcDNA5. The cells were harvested at 48 h post-transfection (pt), and western blotting was done to check the expression of the different CHIKV E1 mutant proteins. (**B**) The cells co-transfected with pAGPAT1-HA and the plasmids expressing the CHIKV E1-FLAG protein or its mutants, or the empty vector pcDNA5 (as indicated at the top of individual panels) were fixed 48 h pt, and the proximity ligation assay (PLA) was performed. The red dots indicate the interaction of CHIKV E1-FLAG with AGPAT1-HA. (**C**) The PLA signals per cell from 15 fields were determined and shown as a box and whisker plot. The statistical analysis was done by the ordinary one-way analysis of variance (ANOVA) with Dunnett’s multiple comparisons test, considering the WT CHIKV E1 as the control. (**D**) Huh7 cells were co-transfected with the plasmid expressing the CHIKV E1-FLAG protein and the plasmids expressing the AGPAT1-HA protein (WT) or its mutants, or the empty vector pcDNA5-HA. The cells were harvested 48 h pt, and western blotting was done to check the expression of the different AGPAT1 mutant proteins. (**E**) The cells co-transfected with the plasmid expressing the CHIKV E1-FLAG protein and the plasmids expressing the AGPAT1-HA protein or its mutants, or the empty vector pcDNA5-HA (as indicated at the top of individual panels) were infected with CHIKV (MOI 1) 24 h pt, fixed 24 h post-infection, and the PLA was performed. The red dots indicate the interaction of CHIKV E1 with AGPAT1-HA. (**F**) The PLA signals per cell from 15 fields were determined and shown as a box and whisker plot. The statistical analysis on data presented at mean ± SD was performed using the ordinary one-way ANOVA with Dunnett’s multiple comparisons test, considering the WT AGPAT1-HA as the control. ***P < 0.001, or ns = not significant.

**Fig 7:**
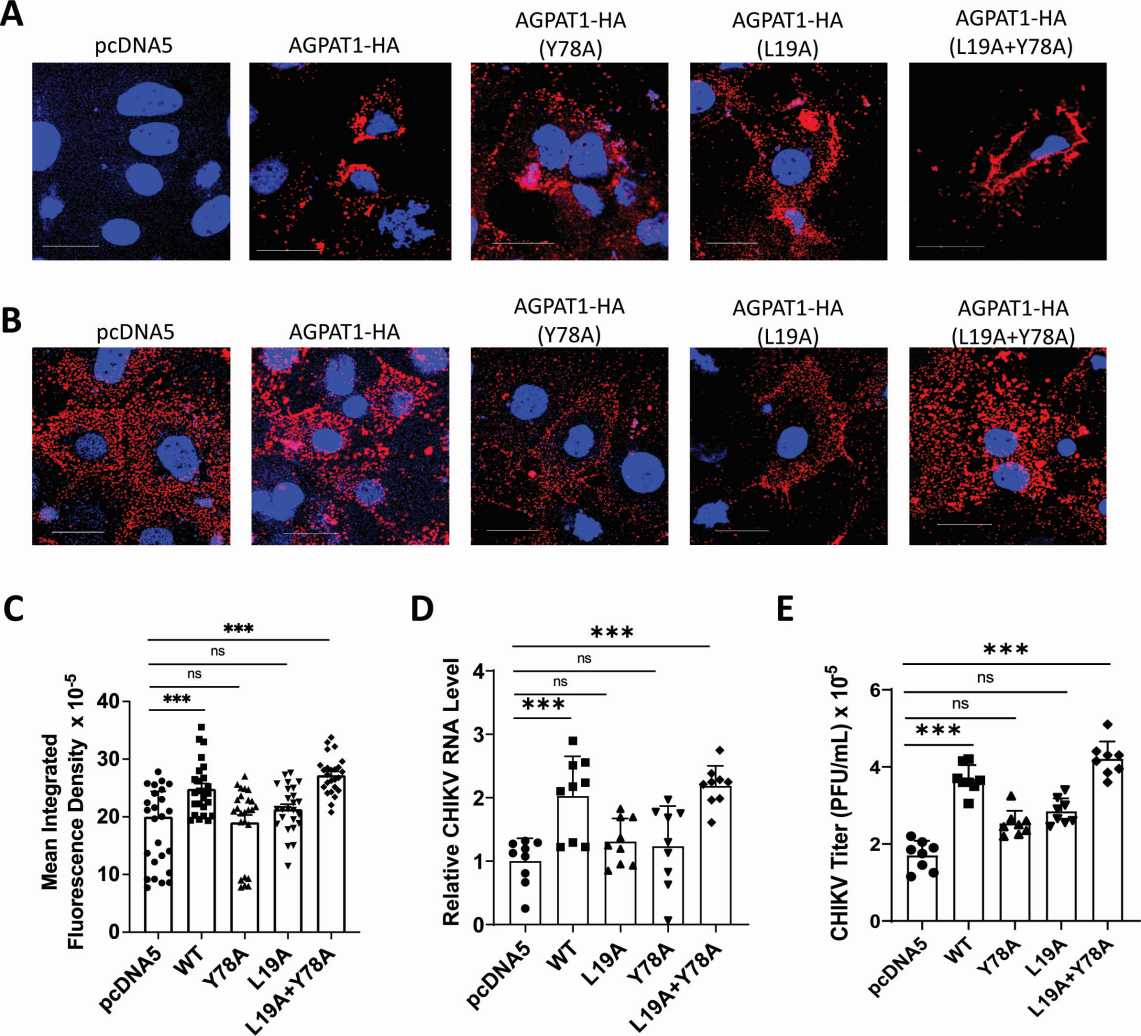
CHIKV binding and uptake in Huh7 cells ectopically expressing the AGPAT1 mutant proteins. (**A**) Huh7 cells were transfected with the plasmids expressing AGPAT1-HA or the mutant proteins, or the empty vector pcDNA5. The non-permeabilized cells were blocked and then stained with the rabbit monoclonal anti-HA antibody, followed by fixing and staining with anti-rabbit Alexa Fluor-568 antibody. The cells were imaged to examine the expression of the ectopically produced AGPAT1-HA or its mutants on the plasma membrane. (**B**) Huh7 cells were transfected with the plasmids expressing AGPAT1-HA or the mutant proteins, or the empty vector pcDNA5. The cells were washed at 48 h pt and incubated with CHIKV (MOI 50) on ice for 1 h and stained with CHIKV anti-E2 antibody to study the virus binding by microscopy. (**C**) The MIFD (25 ROI) data of the transfected Huh7 cells stained as above for the CHIKV binding are presented from three biological replicates. (**D**) Huh7 cells were transfected with the plasmids expressing AGPAT1-HA or the mutant proteins, or the empty vector pcDNA5. The cells were incubated 48 h later with CHIKV (MOI 3) for the virus uptake assay. The relative CHIKV RNA levels are presented, where the CHIKV RNA level in cells transfected with pcDNA5 was taken as 1. The data from five biological replicates and two technical replicates are shown. (**E**) Huh7 cells were transfected with the plasmids expressing AGPAT1-HA or the mutant proteins, or the empty vector pcDNA5. The cells were infected 48 h later with CHIKV (MOI 3) and incubated at 37°C. The virus titers in the culture supernatant at 6 h pi are presented. The data from four biological replicates and two technical replicates are shown. The statistical analysis on data shown as mean±SD was done by the one-way ANOVA with Dunnett’s multiple comparisons test, considering the pcDNA5-transfected cells as the control. ***P < 0.001, or ns = not significant.

Critical residues involved in the AGPAT1 interaction with the E1–E2 dimer were confirmed via *in silico* per-residue energy decomposition analysis (Fig. S7). From these residues, the alanine scanning was performed to identify the single and pair-wise amino acids of both proteins, where a change to alanine will disturb the stability of the complex without affecting the protein’s native structure (Fig. S7). Based on computational interaction energy and interaction type analysis, seven mutations from the E1 subunit and three mutations from AGPAT1 were selected from an initial set of 15 and 6 mutations, respectively, for further *in vitro* analysis (Fig. S7). Western blotting established the ectopic expression of the mutant proteins in the transfected Huh7 cells (Fig. 6A and D). The wild-type and the mutant AGPAT1 proteins were located on the plasma membrane as seen by confocal microscopy (Fig. 7A; Fig. S8A). The expression levels of the mutant proteins did not differ significantly from that of the wild-type AGPAT1 (Fig. S8B).

While CHIKV E1 mutants P74A, R206A, and F192A + R206A showed no difference in their interaction with AGPAT1 as shown by the PLA signals, mutants F192A, N216A, Q218A, and F192A + N216A showed a significantly reduced (*P* < 0.001) interaction compared to the control (Fig. 6B and C). For AGPAT1, Y78A and L19A mutations significantly reduced (*P* < 0.001) its interaction with CHIKV E1 in the CHIKV-infected cells, while the double mutant L19A + Y78A showed no difference compared to the control (Fig. 6E and F). These data confirm the interaction of AGPAT1 with CHIKV E1 and validate the *in silico* findings on critical amino acids involved in the interaction.

### CHIKV binding and uptake in Huh7 cells ectopically expressing the AGPAT1 mutant proteins

CHIKV uptake and replication were studied in Huh7 cells transfected with the plasmids expressing the mutant AGPAT1-HA proteins. The expression of the ectopically expressed mutant proteins was validated by staining the non-permeabilized cells with HA antibody (Fig. 7A). The CHIKV binding, measured as MIFD on CHIKV-incubated stained cells, was enhanced (*P* < 0.001) in the cells expressing the WT AGPAT1-HA (Fig. 7B and C). However, this enhancement was lost in the mutants Y78A and L19A. Interestingly, the double mutant Y78A + L19A showed an enhanced (*P* < 0.001) CHIKV binding compared to the single mutants, and this was similar to that seen for the WT AGPAT1-HA. Along the same lines, CHIKV uptake (Fig. 7D) and viral replication (Fig. 7E) were significantly enhanced in the cells expressing the WT AGPAT1-HA protein. Again, this enhancement was lost in the mutants Y78A and L19A. Notably, the AGPAT1 double mutant L19A + Y78A showed an enhanced CHIKV uptake and replication compared to the single mutants, and this was similar to that seen for the WT AGPAT1-HA.

A plausible explanation for why the double AGPAT1 mutant protein (L19A + Y78A) would phenocopy WT AGPAT1-HA may be rooted in the concept of cooperative or compensatory mutation (35). While the single mutations at L19A or Y78A might individually disrupt critical contact points or structural features necessary for AGPAT1 to interact effectively with E1 or CHIKV, their combination could induce a compensatory conformational change or restore the local structural integrity, thereby re-enabling the native-like mode of binding.

The data presented above ratify the earlier finding on protein-protein interaction (Fig. 5) and demonstrate the role of AGPAT1 in CHIKV binding and uptake in Huh7 cells.

### CHIKV and AGPAT1 co-localize in the early endosomes

**Fig 8:**
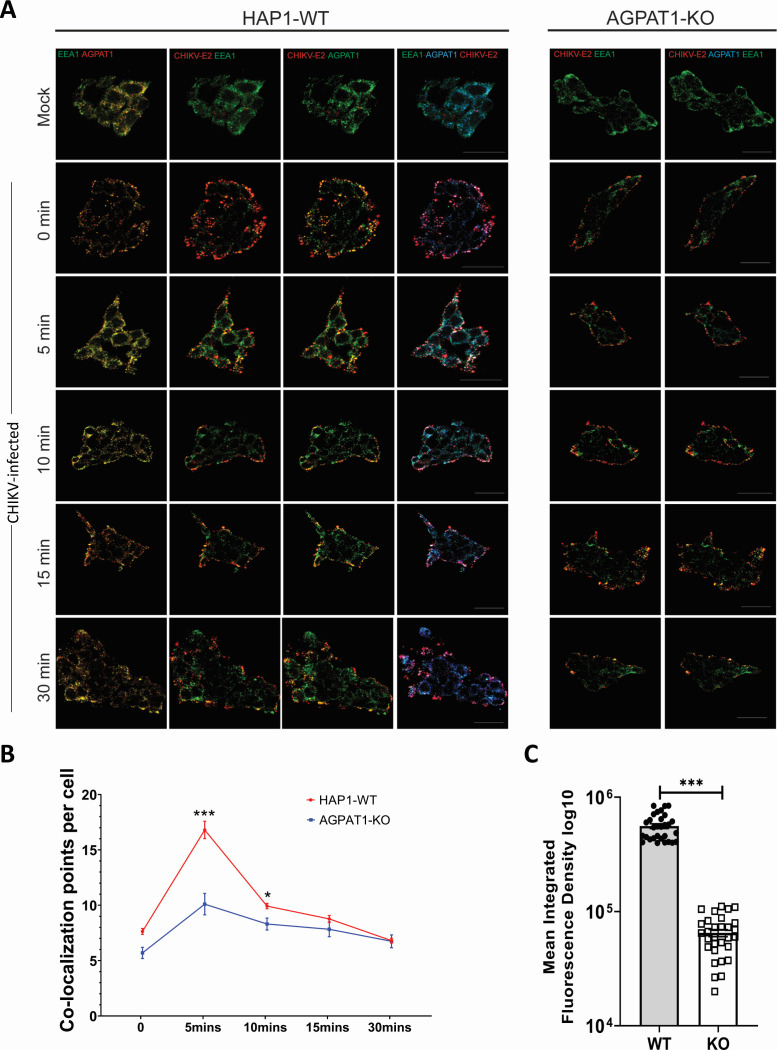
CHIKV and AGPAT1 co-localize in the early endosomes. (**A**) HAP1-WT and AGPAT1-KO cells were infected with CHIKV (50 MOI). The cells were fixed at different times post infection (pi) with 4% para-formaldehyde and permeabilized by 0.3% Tween-20 before staining with anti-CHIKV E2 antibody for CHIKV, anti-AGPAT1 antibody for AGPAT1, and anti-EEA1 antibody for early endosome protein EEA1, followed by imaging. CHIKV-E2 was tagged with Alexa 647 (anti-mouse), AGPAT1 with Alexa 488 (anti-rabbit), and EEA1 with Alexa 568 (anti-goat). (**B**) Graphical representation of the quantification of colocalization of AGPAT1-CHIKV E2 and EEA1. The co-localized dots for these three proteins were observed as white dots resulting from the merger of red, green, and blue colors, as seen in the extreme right columns. (**C**) Graphical representation of the CHIKV binding to HAP1-WT and AGPAT1-KO cells as the MIFD (30 ROI) values at 0 min pi is provided. ***P < 0.001.

Internalization into EEA1-positive early endosomes is direct evidence that the protein-virus complex is actively endocytosed. This is a major hallmark of the receptor role for the host protein as opposed to mere attachment, which may not result in productive endocytosis. To examine the role of AGPAT1 as a CHIKV receptor, confocal microscopy was used to study if the AGPAT1-CHIKV complex localized to the early endosomes (Fig. 8A; Figs. S9 and S10). We used HAP1 WT and AGPAT1 KO cells for this experiment. At the zero-time point, as seen before, CHIKV binding (indicated by red fluorescence for CHIKV E2) is lower in the KO cells compared to HAP1-WT cells (Fig. 8C). In the WT cells at 5-min post-infection, a significant fraction of CHIKV-E2 colocalizes with AGPAT1 and the early endosome marker EEA1, indicative of internalization of the viral protein and the receptor in the early endosomes. In the AGPAT1 KO cells, a significantly lower fraction of CHIKV E2 colocalizes with EEA1, highlighting the essential role of the AGPAT1 protein in virus internalization (Fig. 8A and B).

### Role of AGPAT1 in binding and uptake of Ross River virus (RRV)

**Fig 9:**
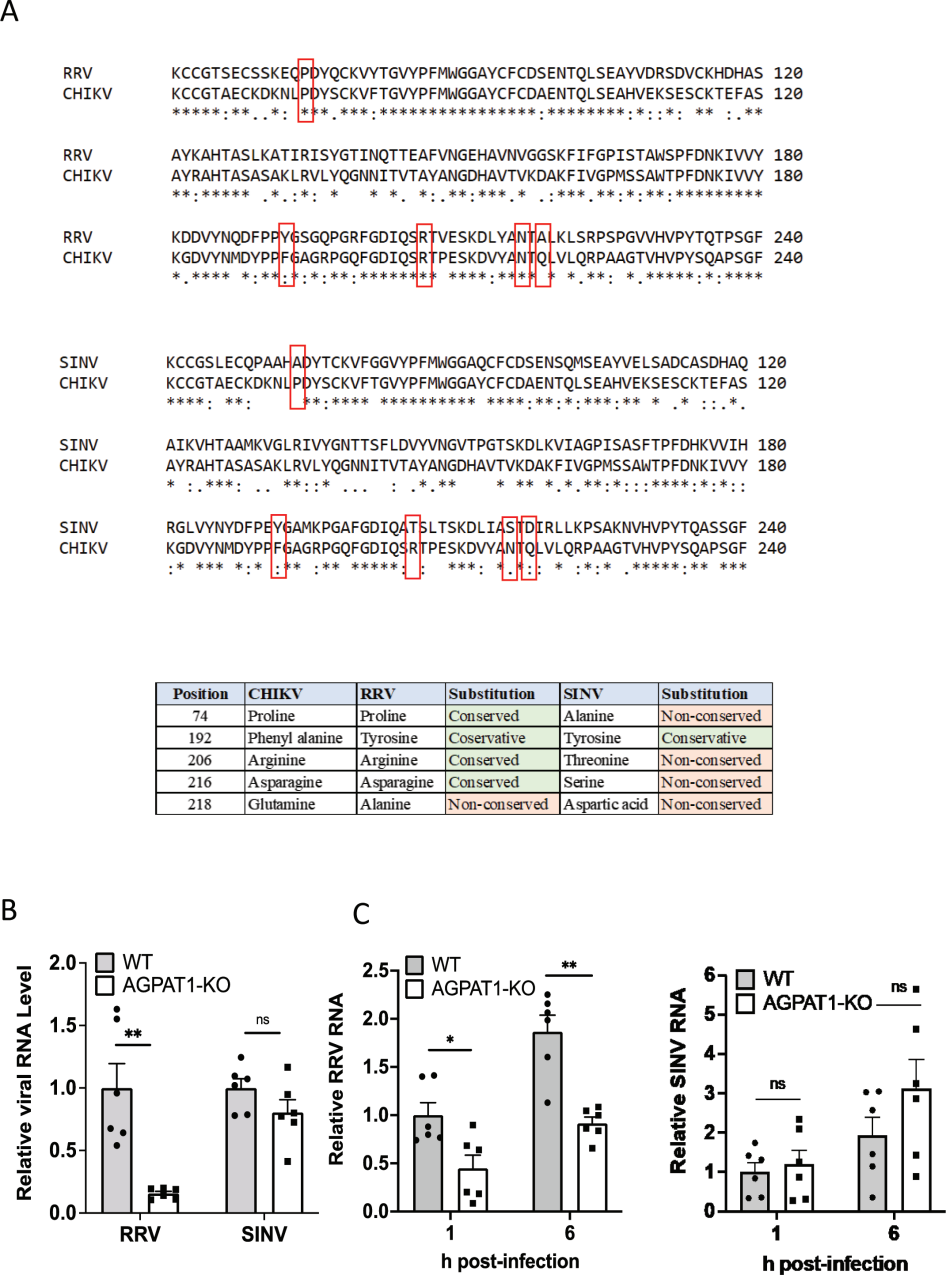
Role of AGPAT1 in RRV binding and uptake in Huh7 cells. (**A**) The amino acid sequence of the CHIKV E1 protein (GenBank ID JF274082) was aligned with that of the RRV E1 protein (GenBank ID GQ433359.1) and SINV E1 protein (GenBank ID V01403.1) using the program Clustal Omega. Amino acid numbering is provided at the right. Conserved amino acids are marked with an asterisk (*) and conservative changes as (:), whereas semi-conservative changes are marked as (.) under the aligned amino acid sequence. The amino acids involved in CHIKV E1 interaction with AGPAT1 are boxed. The table lists the amino acid substitutions between the CHIKV E1, RRV E1, and SINV E1 proteins and identifies the nature of the change. (**B**) The KO and WT HAP1 cells were incubated with RRV and SINV on ice for 30 min at 5 MOI for the virus binding assay. The relative viral RNA levels are presented where the viral RNA level in the WT cells was taken as 1. The data from three biological replicates and two technical replicates are shown. (**C**) The WT and AGPAT1 KO HAP1 cells were incubated with RRV or SINV (MOI 1) for 1 h on ice for the virus uptake assay. The relative viral RNA levels are presented where the RNA level in the WT cells at 1 h was taken as 1. The data from three biological replicates and two technical replicates are shown. **P < 0.01, or ns = not significant.

RRV, an alphavirus, shows significant conservation of the E1 amino acids involved in CHIKV E1 binding with AGPAT1 (Fig. 9A). However, Sindbis virus (SINV), another alphavirus, shows little conservation of these E1 residues. Interestingly, RRV binding to AGPAT1 KO HAP1 cells was reduced by 85% compared to the WT cells (*P* < 0.01), whereas SINV binding was not affected (Fig. 9B). Similarly, RRV uptake in the KO cells was lower (*P* < 0.01) by 56% than in the WT cells; however, SINV uptake was not affected (Fig. 9C).

### Role of AGPAT1 in CHIKV binding in muscle cells

**Fig 10:**
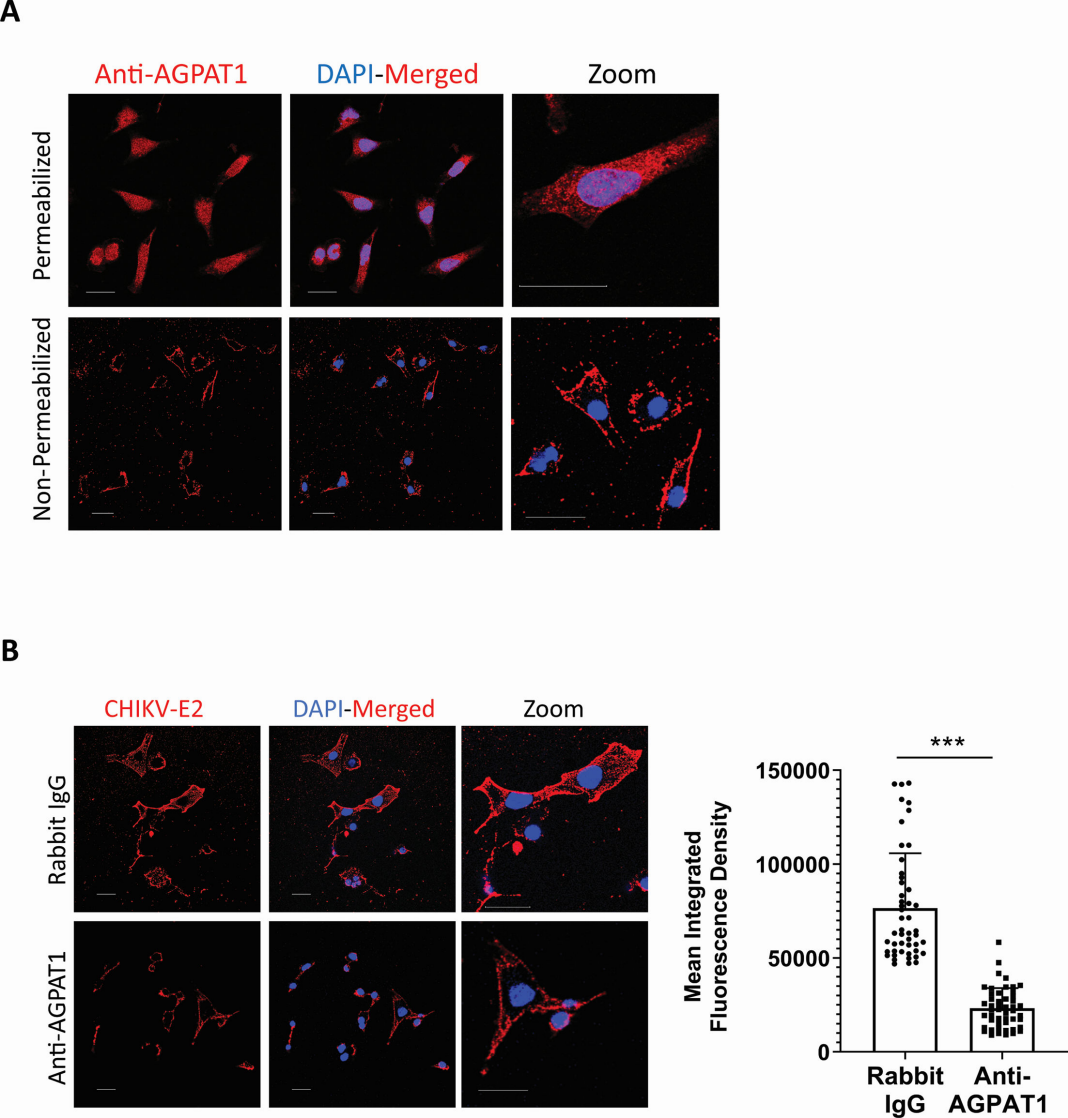
Role of AGPAT1 in CHIKV binding in ERMS cells. (**A**) ERMS cells were permeabilized with 0.3% Tween-20 or were not permeabilized. The cells were then incubated with rabbit AGPAT1 antibody, followed by fixing the cells in 2% paraformaldehyde and staining with Alexa Fluor-568 antibody. The nuclei were stained with DAPI, and the cells were imaged. (**B**) ERMS cells were incubated on ice with rabbit anti-AGPAT1 polyclonal antibody or rabbit IgG (isotype control) for 30 min at 10 µg/mL concentration, followed by incubation with CHIKV (MOI 50) on ice for 30 min. The cells were then incubated with CHIKV anti-E2 mouse monoclonal antibody on ice for 30 min and washed with ice-cold PBS. The cells were stained with Alexa Fluor-647 anti-mouse antibody for 30 min. The nuclei were stained with DAPI, and the cells were imaged. The MIFD (50 ROI) is shown as mean ± SD in the right panel. ***P < 0.001.

**Fig 11:**
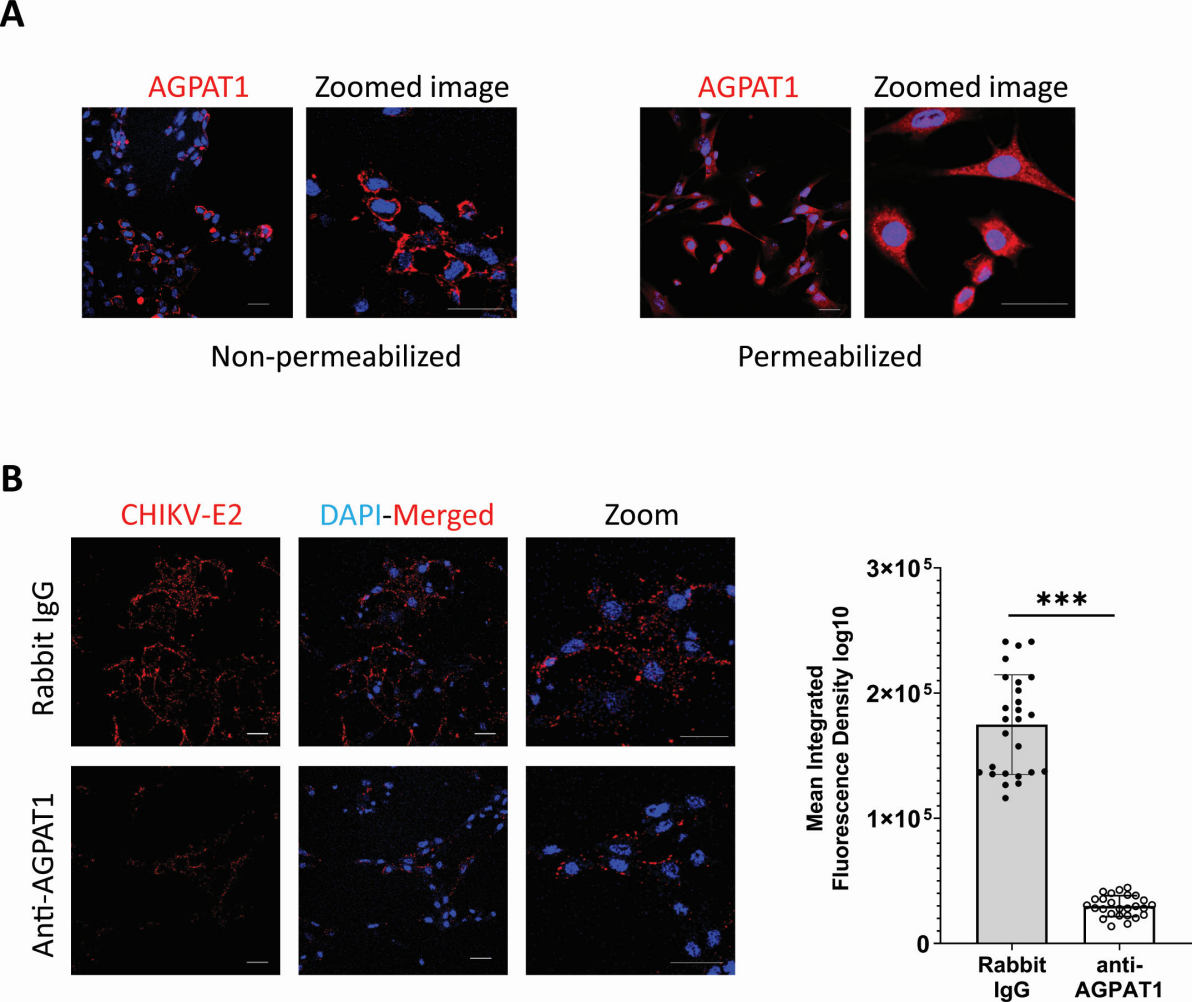
CHIKV binding in the primary mouse fibroblast cells. (**A**) The primary mouse fibroblast cells were permeabilized with 0.3% Tween-20 or were not permeabilized. The cells were then incubated with rabbit AGPAT1 antibody, followed by fixing the cells in 2% paraformaldehyde and staining with Alexa Fluor-568 antibody. The nuclei were stained with DAPI, and the cells were imaged. (**B**) The primary mouse fibroblast cells were incubated on ice with rabbit anti-AGPAT1 polyclonal antibody or rabbit IgG (isotype control) for 30 min at 10 µg/mL concentration, followed by incubation with CHIKV (MOI 50) on ice for 30 min. The cells were then incubated with CHIKV anti-E2 mouse monoclonal antibody on ice for 30 min and washed with ice-cold PBS. The cells were stained with Alexa Fluor-647 anti-mouse antibody for 30 min. The nuclei were stained with DAPI, and the cells were imaged. The MIFD (25 ROI) is shown as mean ± SD in the right panel. ***P < 0.001.

CHIKV replication in skeletal muscle cells is a critical mediator of CHIKV pathogenesis (36–40). We, therefore, studied the role of AGPAT1 in CHIKV infection in ERMS cells of the human skeletal muscle lineage. Here also, AGPAT1 was present on the plasma membrane (Fig. 10A). AGPAT1 antibodies caused a ~70% reduction (*P* < 0.001) of CHIKV binding to ERMS cells based on MIFD, suggesting that CHIKV uses AGPAT1 to bind to ERMS cells also (Fig. 10B). Importantly, in the primary mouse fibroblast cells, AGPAT1 was located on the plasma membrane (Fig. 11A). Furthermore, AGPAT1 antibody significantly inhibited CHIKV binding to the primary mouse fibroblast cells, decreasing ~84% (*P* < 0.001) based on the MIFD quantification (Fig. 11B).

## DISCUSSION

Virus receptors determine the host specificity and cell tropism, and the knowledge of the virus-host receptor interactions could help develop novel antivirals targeting the very first step in the virus infection of the host cell. So far, Mxra8, Prohibitin, and CD147 proteins have been described as CHIKV receptors in mammalian hosts. Of these, the Mxra8 receptor for CHIKV has been extensively characterized (9–11, 27, 41–43). Mxra8 had a role in CHIKV infection in mice and several human cell lines (23, 27). However, cells such as HEK-293T and Huh7 (27) or HAP1 (24), lacking the surface expression of MXRA8, are susceptible to CHIKV infection, suggesting that additional host proteins serve as CHIKV receptors in mammalian cells. We have identified AGPAT1 as a CHIKV receptor on human cell lines such as Huh7, ERMS, and HAP1. Importantly, these cells lack MXRA8 expression on their plasma membrane (Fig. S11). Thus, the identification of AGPAT1 as an additional receptor is a significant advancement in understanding the CHIKV pathogenesis in humans, especially in the context of CHIKV infection of cells lacking MXRA8. 

AGPAT1 is an enzyme involved in the biosynthesis of phosphatidic acid, a key precursor for the formation of structural phospholipids and diacylglycerol in cells. In cell physiology, AGPAT1 regulates the differentiation of skeletal muscle cells by influencing myogenic transcription factors and the actin cytoskeleton, and its loss leads to metabolic, reproductive, and neurological abnormalities due to disrupted phospholipid homeostasis. AGPAT1’s enzymatic activity is crucial for maintaining membrane structure and function across multiple organ systems (32, 44, 45).

In the context of virus replication, AGPAT1 is co-opted by positive-strand RNA viruses such as hepatitis C virus (HCV) and SARS-CoV-2, which rely on AGPAT1-derived phosphatidic acid for the formation of double-membrane vesicles—specialized replication organelles necessary for efficient viral genome amplification (46). Experimental depletion of AGPAT1 impairs the replication of these viruses by disrupting vesicle biogenesis. In contrast, viruses like dengue virus (DENV) actively downregulate AGPAT1, creating a cellular environment more favorable for their replication; AGPAT1 depletion increases DENV replication, likely by altering cellular lipid composition to a pro-viral state (46). Thus, AGPAT1 activity and its regulation are tightly linked to both normal cell physiology and the replication strategies of diverse viruses, although the protein has not been reported to have a direct role in the virus life cycle. Thus, this is the first report showing a direct role for AGPAT1 in the virus life cycle as a novel CHIKV receptor on mammalian cells.

Different methods have been employed to study the CHIKV receptor. For example, Prohibitin was identified as binding to CHIKV using a virus overlay protein binding assay (VOPBA) on human microglial CHME-5 cells (28). On the other hand, Mxra8 was identified by CRISPR-Cas9 screening for CHIKV infection in the mouse fibroblast NIH3T3 cells (27). The Strep-tagged CHIKV envelope protein complex was used to identify CD147 protein in HEK-293T cells (29). We employed a pull-down method to detect the Huh7 plasma membrane proteins directly binding to CHIKV virions. The mass spectrometry identified several proteins, including Prohibitin, which validated the method used in our study. However, we did not detect MXRA8 in our experiments. This may be related to the low level of MXRA8 or its absence in the Huh7 cell membrane (Fig. S11) compared to the mouse fibroblast NIH 3T3 cells where Mxra8 was identified as a CHIKV receptor (27). The CRISPR-Cas9 screening for CHIKV infection in the mouse fibroblast NIH3T3 cells did not pick up Prohibitin or Agpat1 (27). For AGPAT1, this may be related to its absence on the NIH3T3 cell membrane (Fig. S11).

AGPAT1 is primarily located in the ER membranes (33). For AGPAT1 to act as a virus receptor, it must be located on the plasma membrane. Our study demonstrated the presence of AGPAT1 on the plasma membrane of Huh7, HAP1, and ERMS cells by confocal microscopy on non-permeabilized cells and western blotting of the plasma membrane fraction of Huh7 cells. In the mouse myoblast C2C12 cells (Fig. S11), AGPAT1 was found on the plasma membrane as reported before (44).

Using the pull-down mass spectrometry method, we identified AGPAT1 from Huh7 cells as a protein binding with CHIKV virions. The interaction of AGPAT1 with CHIKV was verified using co-immunoprecipitation, while the binding of CHIKV with AGPAT1 was demonstrated on Huh7, HAP1, and ERMS plasma membrane by confocal microscopy and other methods. Antibody to AGPAT1 inhibited CHIKV binding to the plasma membrane and reduced virus uptake in Huh7 and ERMS cells. The role of AGPAT1 in CHIKV binding and uptake was further confirmed in HAP1 cells, where CHIKV binding, uptake, and replication were significantly reduced in AGPAT1 KO cells. Ectopic expression of AGPAT1 in the KO cells rescued the reduced CHIKV binding, uptake, and replication; a huge enhancement (2,300%) of CHIKV uptake was seen in the rescued cells. Furthermore, the ectopic expression of AGPAT1 on Huh7 plasma membrane enhanced CHIKV binding to cells, and this enhancement was not seen with AGPAT1 mutants. These data demonstrate that AGPAT1 has an important role in CHIKV binding and uptake in Huh7 and HAP1 cells. Importantly, a role for AGPAT1 in CHIKV binding in ERMS cells, as well as primary mouse muscle fibroblast cells, was also shown.

Colocalization of a virus–protein complex with the early endosome marker EEA1 is widely used as experimental evidence that a virus–protein complex is actively internalized via receptor-mediated endocytosis, supporting its designation as a virus receptor. For example, colocalization of CHIKV and MXRA8 with EEA1 was crucial evidence in establishing MXRA8’s functional role as an entry receptor, not just an attachment factor (41). Colocalization of human coronavirus NL63 particles with EEA1 by confocal microscopy showed that entry depends on internalization into early endosomes, triggered by the ACE2 receptor engagement (47). Colocalization of mouse polyomavirus with EEA1 was used to show that the majority of internalized virions enter EEA1-positive early endosomes—evidence for productive endocytosis (48). Similarly, internalization of hepatitis C virus capsid-like particles in Huh7 cells, analyzed by EEA1 colocalization, was used to measure productive endocytic entry (49). Our data showing the role of AGPAT1 in CHIKV binding and uptake and the localization of the CHIKV E2-AGPAT1 proteins, together with EEA1, support the role of AGPAT1 as a CHIKV receptor.

CHIKV is an enveloped virus projecting the viral envelope proteins E1 and E2 on its outer surface (34). The heterodimer of the E1 and E2 proteins is present as a trimer on the virion envelope (34, 50). Our *in silico* studies predicted the interaction of AGPAT1 with the CHIKV E1–E2 dimer at the E1 surface. This was validated by confocal microscopy, showing the colocalization of CHIKV E1 protein with AGPAT1 in Huh7 cells. The *in silico*-predicted critical amino acids at the E1 surface, majorly interacting with AGPAT1, were validated using the mutant proteins. Studies using site-directed mutant viruses would greatly help in further confirming these findings.

While no information is available on how Prohibitin interacts with CHIKV, mammalian MXRA8 was shown to interact with CHIKV E1 as well as E2 protein (9). Here, we have demonstrated AGPAT1 interaction with CHIKV E1 and identified the key amino acids of the proteins involved. Interestingly, some of these residues of CHIKV E1 were also engaged in CHIKV interaction with mammalian MXRA8 (9). It may be noted that the avian MXRA8 was shown to interact with the E1 protein of Western equine encephalitis virus, yet another alphavirus (11).

AGPAT1 is a 283-amino acid-long membrane protein. Its transmembrane (TM) domains and the location of cytosolic and luminal residues have not been clearly established, and these have all been reported based on prediction using different modeling algorithms. Yamashita et al. (51) predicted three or four TM domains using different methods. The authors note that based on these predictions, the conserved acyltransferase motifs thought to be essential for catalytic activity would be separated by TM3; as a consequence, motifs I, II, and III would be located on the cytosolic and lumenal sides of the ER membrane, respectively. This predicted topology is difficult to reconcile with the expectation that the acyltransferase motifs act in concert to convert lysophosphatidic acid (LPA) and acyl-CoA to phosphatidic acid (PA). They hypothesized that the loop carrying motifs II and III may penetrate into the membranes so as to reach the outer leaflet of the membrane. Interestingly, UniProt (Q99943) only shows 2 TM domains in human AGPAT1, involving residues 38–58 and 128–248. Accordingly, AGPAT1 residues predicted as important for interacting with CHIKV fall in luminal/cytoplasmic topology. Thus, it is likely that AGPAT1 TM domains are not fully integrated inside the plasma membrane and instead are exposed, at least partly, on the outer surface of the plasma membrane.

Limitations of computational predictors contribute to discrepancies where terminal residues and dynamic membrane interfaces are particularly hard to model accurately. Consequently, residues at the membrane-water boundary may be exposed, despite their bioinformatic transmembrane assignment (52). For example, the transmembrane helix of glycophorin A exposes specific hydrophobic residues to the membrane core, promoting helix-helix dimerization (53, 54). In *Escherichia coli*, lipoprotein RcsF adopts a transmembrane orientation with the lipidated N-terminus on the cell surface and the folded C-terminal domain in the periplasm (55). Protein-specific motifs and non-canonical membrane insertion sequences may cause predicted transmembrane segments to behave unconventionally and remain accessible extracellularly. For example, outer membrane proteins Hop and HorB in *Helicobacter pylori* feature conserved C-terminal motifs that, despite being predicted as transmembrane-like, are experimentally shown to be surface-exposed adhesins (56).

Applying these insights to AGPAT1, it is likely that its predicted transmembrane domains are at least partially situated near the outer leaflet membrane interface, not fully embedded, thus remaining accessible for virus binding, allowing AGPAT1 to function as a CHIKV receptor. Only a crystal structure of AGPAT1 will conclusively show the correct topology of specific CHIKV-interacting residues.

Several studies have demonstrated the critical role of different lipid molecules in alphavirus entry, membrane organization, RNA synthesis, and virion assembly (57–60). AGPAT1 catalyzes the transformation of LPA into PA, which serves as an essential precursor for the synthesis of other phospholipids and triacylglycerols (61). In addition, PA regulates endocytosis and vesicular trafficking processes in the cell (62, 63), which may impact CHIKV entry. Therefore, further experiments should be undertaken to evaluate the contribution of AGPAT1’s enzymatic activity to CHIKV replication.

CHIKV has an extensive host range, infecting cells of multiple origins in human and mosquito hosts. It is, therefore, likely that the virus uses as a receptor a protein ubiquitously present on cells of different origins. Alternatively, the virus may use different proteins on different cells. The latter seems to be the case as AGPAT1, Mxra8, Prohibitin, and CD147 have been identified as CHIKV receptor proteins on different cell types. Again, these may not be the only proteins the virus uses as a receptor, and therefore, the search for additional proteins as CHIKV receptors must continue. We have shown AGPAT1’s presence and role in CHIKV replication in several transformed human and mouse cell lines and the primary mouse fibroblast cells. HAP1 is a human myeloid leukemia-derived cell line. This raises the possibility that AGPAT1 is also expressed in primary human myeloid cells, such as monocytes, macrophages, and dendritic cells, that are involved in the virus-triggered inflammatory response. This needs further investigation as it has a bearing on virus pathogenesis in humans.

## MATERIALS AND METHODS

### Cells and viruses

The Vero, BHK-21, Huh7, and NIH 3T3 cell lines were obtained from the National Centre for Cell Sciences (NCCS) cell repository. The human ERMS (RD-CCL-136-ATCC) and C2C12 (CRL-1772) were obtained from the ATCC, USA. The minimum essential medium, Eagle (MEM) (HiMedia, India; AL0475), was used to cultivate Vero and BHK-21 cells. Dulbecco’s modified Eagle medium (DMEM) (HiMedia, India; AL007A) was used to culture Huh7, ERMS, C2C12, and mouse primary muscle fibroblast cells. HAP1 cells were cultured using Iscove’s modified Dulbecco’s Medium (IMDM) (HiMedia, India; AL070S). All the media were supplemented with 10% fetal bovine serum (FBS) (Gibco; 10270106) and 1× penicillin-streptomycin-glutamine (PS) solution (HiMedia, India; A001A). The cells were grown at 37°C under a 5% CO_2_ atmosphere. The primary mouse muscle fibroblast cells were prepared using the method described by Kumar et al. (64).

The IND-06-Guj (JF274082; GenBank) was used in this study. RRV and SINV were obtained from the World Reference Center for Emerging Viruses and Arboviruses, The University of Texas Medical Branch, USA.

### Purification of Chikungunya virus

The sucrose gradient-purified CHIKV was used in this study. BHK-21 cells were cultured in a T175 flask, infected with CHIKV for 1 h at 0.1 MOI, and incubated at 37°C for 36 h. The culture supernatant was collected, and the virus was precipitated by 8% PEG-8000 at 4°C. The virus pellet was suspended in the NTE buffer (150 mM NaCl, 50 mM Tris, and 5 mM EDTA, pH 8.0) and over-layered on a discontinuous 30%–60% sucrose gradient. The virus was ultracentrifuged overnight at 4°C in a SW28Ti rotor (Beckman Coulter) at 26,000 rpm. The virus layer collected from the gradient interphase was overlaid on a 20% sucrose bed (2 mL) and pelleted at 4°C for 6 h using the SW41Ti rotor (Beckman Coulter) at 39,000 rpm. The virus pellet was re-suspended in MEM and stored in small aliquots at −80°C. The virus purity was checked by SDS-PAGE, and its titer was determined by plaque assay on Vero cells.

### Plaque assay for CHIKV titration

Vero cells were plated overnight in a six-well plate to obtain 70% confluency. Serial dilutions of the CHIKV sample were made in MEM, and 200 µL of virus from different dilutions was used to infect the cells at 37°C for 1 h. The wells were then washed and overlaid with 1% agarose in MEM. The plates were incubated at 37°C for 36 h to obtain the plaques. The cell monolayers were fixed with 4% formalin for 2 h and stained with 0.2% crystal violet for 1 h. The plates were then washed with water and air-dried. The plaques formed were counted, and the virus titer was calculated as plaque-forming unit (PFU)/mL using the dilution factor.

### Purification of the plasma membrane proteins

The Huh7 cell monolayer grown in T175 flasks was washed with 1× PBS and scraped in the same. A Dounce homogenizer (100 cycles) was used to lyse the cells with the homogenization buffer containing the protease inhibitor cocktail. The plasma membrane proteins were isolated using a plasma membrane isolation kit (ab65400; Abcam). The total cell lysate and the cytosolic and plasma membrane protein fractions were run on an SDS-PAGE gel and western-blotted. E-cadherin was used as a marker for the plasma membrane protein fraction, GAPDH as a marker for the cytoplasmic proteins, and Calreticulin was used to detect the ER membrane contamination in the preparation. The following antibodies were used for the western blotting: rabbit monoclonal anti-E-Cadherin antibody (CST 3195; Cell Signaling Technologies), rabbit polyclonal GAPDH antibody (GTX100118; GeneTex), rabbit polyclonal anti-Calreticulin antibody (2891; Cell Signaling Technologies), and mouse polyclonal AGPAT1 antibody (ab67018; Abcam).

### Pull-down of plasma membrane proteins with the agarose-conjugated CHIKV

Affinity purification coupled with mass spectrometry was employed to identify the CHIKV-binding cellular proteins (29). Briefly, the sucrose gradient-purified CHIKV (150 µg) was immobilized on Amino-Link agarose beads (Thermo Fisher Scientific) by amine linkage chemistry using sodium cyanoborohydride. The Pierce co-immunoprecipitation kit (Thermo Fisher Scientific) was used to incubate the CHIKV beads with the Huh7 plasma membrane proteins in 1× PBS, 5% octyl-β-D-glucopyranoside. The beads were washed with 1× PBS, 5% octyl-β-D-glucopyranoside, and the bound proteins were eluted using a low pH buffer (Glycine, pH 2.8), followed by neutralization with 1.5 M Tris pH 9. The eluted proteins were run on an SDS-PAGE, followed by gel extraction. The proteins were trypsin (V5280, Promega) digested and subjected to mass spectrometry using an electrospray ionization mass spectrometer: SCIEX TripleTOF 5600+. The mass spectrometry output was analyzed through MASCOT and Protein Pilot to identify the proteins.

### Immunostaining for localization of AGPAT1 on the plasma membrane

The cells were grown overnight at 37°C on coverslips (Bluestar) placed in a 24-well plate (Corning). The coverslips were washed with chilled 1× PBS and blocked with 5% BSA on ice. For localization of AGPAT1, the cells were incubated on ice for 1 h with rabbit polyclonal anti-AGPAT1 (ab235328; Abcam). The cells were then fixed with 2% formaldehyde for 20 min at room temperature, followed by 30 min incubation of anti-rabbit Alexa Fluor-568 antibody (A11011; Invitrogen) for staining AGPAT1. The coverslips were mounted on Prolong Gold antifade with DAPI (P36935; Invitrogen) and observed under the Leica SP8 confocal microscope. The figures show representative images from multiple experiments.

### Immunostaining of proteins in the permeabilized mammalian cells

The cells grown overnight at 37°C on coverslips (Bluestar) placed in a 24-well plate (Corning) were washed twice with PBS and fixed with 2% paraformaldehyde for 20 min at room temperature. For permeabilization, the cells were incubated with 0.3% Tween-20 or 4% paraformaldehyde and blocked with 5% BSA for 1 h at room temperature. The cells were stained for 2 h at room temperature with rabbit polyclonal anti-AGPAT1 antibody (ab235328; Abcam) for AGPAT1, mouse monoclonal anti-E1 (GTX135187; R&D systems) for CHIKV E1, goat polyclonal anti-EEA1 (ab206860; Abcam) for EEA1, rabbit monoclonal anti-HA (3724; Cell Signaling Tech) or mouse monoclonal anti-HA (2367; Cell Signaling Tech) for AGPAT1-HA, rabbit monoclonal anti-FLAG (14793; Cell Signaling Tech), or mouse monoclonal anti-FLAG (91878; Invitrogen) for E1-FLAG. After washing with PBS, the cells were incubated at room temperature for 1 h with anti-mouse Alexa Fluor-647 antibody (4410S; Cell Signaling Tech), anti-rabbit Alexa Fluor-568, anti-rabbit Alexa Fluor-488 antibody (A11008; Invitrogen), or anti-goat Alexa Fluor-647 (A21447; Invitrogen). The coverslips were mounted with Prolong Gold antifade with DNA stain DAPI (Thermo Fisher Scientific) and observed under the Leica SP8 confocal microscope. The images were processed using LasX software (Leica). The figures show representative images from multiple experiments. The scale bar shown in images is 25 μm unless indicated otherwise.

### Structured illumination microscopy

Structured Illumination Microscopy (SIM) imaging was performed using a Zeiss Elyra PS1 microscope equipped with a Plan-Apochromat 63×/1.4 Oil DIC M27 objective. Fluorescence signals were detected with an Andor EMCCD camera. Excitation was performed using 405 nm, 488 nm, and 642 nm lasers at 10% power each. Images were acquired in unidirectional scan mode, with a zoom of 100×. The unprocessed image field of view was 82.54  × 82.54  µm (1,280 × 1,280 pixels), with data collected at 16-bit depth. The figure shows representative images from multiple experiments.

### siRNA-mediated knockdown of AGPAT1

The Mission esiRNA (EHU228041; Merck), containing a pool of siRNAs targeting the human AGPAT1 mRNA, was used to knock down the AGPAT1 protein expression in Huh7 cells. The Mission esiRNA Universal Negative Control #1 (SIC001; Merck) containing the non-targeting pool of siRNAs was used as the control. The esiRNAs at 30 nM concentration were transfected into Huh7 cells using Lipofectamine RNAiMAX reagent (13778075; Invitrogen). At 48 h post-transfection (pt), the knockdown of the protein was quantified by western blotting. GAPDH was used as the control.

### Ectopic expression of protein

The cDNA encoding AGPAT1 or its mutants fused with the HA-tag sequence was cloned in pcDNA5 vector under the CMV promoter. The primers used for the cDNA amplification are described in Table S1. The cDNA encoding CHIKV E1 or its mutants fused with the FLAG-tag sequence was cloned in the pcDNA5 vector under the CMV promoter. The cells were transfected with the expression plasmids using Lipofectamine 3000 transfection reagent (L3000015; Invitrogen). The ectopic expression of the protein was verified by western blotting of the cell lysate with an appropriate antibody. GAPDH was used as the loading control. The figures show representative western blot images from multiple experiments.

### Total RNA isolation, reverse transcription, and quantitative PCR

The total RNA was isolated from the cells using RNAiso Plus (TaKaRa), precipitated by absolute isopropanol, air-dried, and reconstituted in nuclease-free water. The purity and concentration of RNA were determined spectrophotometrically. The cDNA was made using the ImProm-II reverse transcription kit (Promega), which used 50–500 ng RNA and random primers. The cDNA was quantified by qPCR using SYBR green chemistry. The TB Green Premix Ex Taq II (Tli RNaseH Plus) (TaKara) was used as the PCR master mix. The following CHIKV nsP2 primers were used to detect the CHIKV RNA: Forward- GGCAGTGGTCCCAGATAATTCAAG and Reverse- GCTGTCTAGATCCACCCCATAC ATG. The RRV RNA was detected by primers for E1: Forward- GCGACGGT GGATGTCAAGGAG and Reverse- AGCCAGCCCACCTAACCCACTG. The SINV RNA was detected using the following primers for E2: Forward- AAAGGATACTTTCTCCTCGC and Reverse- TGGGCAACAGGGACCATGCA. The human GAPDH RNA was detected by the GAPDH forward primer GGTGAAGGTCGGAGTCAACG and GAPDH reverse primer AGGGATCTCGCTCCTGGAAG. The qPCR was run in the standard curve mode with a melting curve plot under the SYBR green chemistry in a Quantstudio 6 Flex (Applied Biosystems) PCR machine. The data obtained were normalized using the cellular GAPDH levels.

### CHIKV-binding microscopy

For CHIKV localization and binding studies, the cells were incubated for 1 h with CHIKV on ice, followed by blocking with 5% BSA and 1 h incubation with mouse monoclonal anti-E2 (MAB1219; Native Antigen) in 1% BSA on ice. The cells were then fixed with 2% formaldehyde for 20 min at room temperature, followed by 30 min incubation of anti-rabbit Alexa-568 antibody (A11011; Invitrogen) for staining AGPAT1 or anti-mouse Alexa Fluor-647 antibody (4410S; Cell Signaling Technologies) for staining the CHIKV E2 protein. The coverslips were mounted on Prolong Gold antifade with DAPI (P36935; Invitrogen) and observed under the Leica SP8 confocal microscope. The images were processed by the LasX software (Leica). To determine the mean fluorescence intensity density (MIFD), different fields with an ROI of fixed area, having at least 10 cells, were taken into account. The mean of pixel densities from five middle planes was taken from each field before calculating the mean of all the fields. The figures show representative images from multiple experiments.

### CHIKV-binding assay

The monolayer of cells was incubated with CHIKV on ice for 30 min at the indicated MOI to allow the virus binding. The unbound virus was removed by ice-cold PBS wash twice. The cells were then lysed, and total RNA was extracted. The CHIKV binding to cells was determined by the level of CHIKV RNA using qRT-PCR.

### CHIKV uptake and replication assay

The virus uptake was studied by determining the CHIKV RNA levels early during the infection. To this end, the cell monolayer was incubated with CHIKV for 1 h on ice, followed by a wash with chilled PBS. Complete media was added to the cells and incubated at 37°C. The culture supernatants were collected, and the cells were treated briefly with trypsin solution (TCL007; HiMedia, India) before harvesting at different time points post-infection. The total RNA was isolated from the cells, and the virus titers were determined in the culture supernatant. The CHIKV RNA levels were determined by qRT-PCR, and the viral titers were determined by plaque assay. The viral RNA level recorded at 1 h pi was taken as the indicator of the virus uptake and used to calculate the enhancement/reduction in the virus uptake.

### Generation of the AGPAT1 knockout HAP1 cell line

The AGPAT1 knockout (KO) HAP1 cells were generated using the CRISPR-Cas9 technology. The sgRNA sequence 5′-CTCAGCATCAAAGTTAGTAT-3′ was designed using the Broad Institute database for sgRNAs and the CHOPCHOP online predictor and obtained from Genscript as incorporated inside the shuttle vector pSpCas9 BB-2A-Puro (PX459) V2.0. The plasmid containing the sgRNA was transfected into HAP1 cells that were subjected to puromycin selection at 1 µg/mL for six passages. The stable cells were subjected to single-cell clonal selection to obtain a pure AGPAT1 KO cell line. Western blotting was done to establish the absence of the AGPAT1 protein in the KO cells. The figures show representative western blot images from multiple experiments.

### Ectopic expression of AGPAT1 and CHIKV E1 proteins

The ectopic expression of the AGPAT1 and CHIKV E1 proteins was obtained by transfecting the cells with the respective expression plasmid. The expression plasmids were made by inserting the respective cDNAs into the expression vector pcDNA5 (Thermo Fisher Scientific) under the CMV promoter. The plasmid nucleotide sequence was confirmed by Sanger sequencing. The AGPAT1 protein was expressed as a fusion protein with the HA tag, and the CHIKV E1 protein was fused with the FLAG tag. The following primers were used for amplification of the AGPAT1-HA cDNA: Forward, TCAAAGCTTGCCACCATGGATTTG TGGCCAGGGGC (HindIII site underlined) and Reverse, CAGTCTCGAGTTA*TGCATAATC* *CGGAACATCATACGGATA*CCCACCG CCCCCAGGCTTCT (XhoI site underlined, HA-tag sequence in italic). The following primers were used for amplification of the CHIKV E1-FLAG cDNA: Forward, ACCAAGCTTGCCACCATGGGCTACGAACACGTAACAGT GATCCCGAACAC (HindIII site underlined) and Reverse, TCAGGATCCTTA*CTTATCGT* *CGTCATCCTTGTAATC*GTGCCTGCTGAACGACACGCATAGC (BamHI site underlined, FLAG-tag sequence in italic). The protein expression was studied by western blotting, and the figures show representative western blot images from multiple experiments.

### System preparation for the *in silico* studies

The three-dimensional structure of AGPAT1 was taken from the AlphaFold database, and the CHIKV E1-E2 dimer structure was retrieved from the protein data bank (PDB 3N42) (9, 65). To prepare the protein structures, Maestro’s Protein Preparation Wizard module with OPLS4 force field was used; the protein’s missing regions were filled by the prime module, and the preliminary minimization was done by OPLS4 force field. The systems were prepared with the protonation states of the residues at neutral pH as predicted by the PROPKA module in the Protein Preparation Wizard (66, 67).

### Protein-protein docking  

The protein-protein docking of the CHIKV E1-E2 dimer with AGPAT1 was performed using the HDOCK server and AlphaFold 3. A total of 100 docked poses were obtained from HDOCK, and the top five poses of AlphaFold 3 were taken for the analysis. The best poses from the docked models were chosen on the basis of docking score, docking confidence, and cluster size.

### MD simulation

The apo structure of AGPAT1, taken from AlphaFold and the most likely pose obtained from the protein-protein docking (CHIKV E1-E2 dimer with AGPAT1), was used for the MD simulation using the Desmond v6.1 module of the Schrodinger suite. All structures were first minimized with the OPLS4 force field and then solvated with a predetermined TIP3P water solvent model (68). The size of the repeating unit buffered at 12.0 Å distances was determined by placing them all in the orthorhombic periodic boundary conditions (69, 70). Using a steepest-descent integrator for 2,000 steps, structures underwent energy minimization for 500 ps. For the NPT ensemble simulations, the Nose-Hoover chain thermostat and the Martyna-Tobias-Klein barostat were assigned. The RESPA integrator was utilized with a time step of 0.002 ps. The cutoff radius for the short-range Coulombic interactions was 9.0. Bonds were restricted to hydrogen by using the M SHAKE algorithm of Desmond, and the final production run was conducted for 100 ns for the apo structure and one microsecond simulations for the CHIKV E1–E2 dimer complex; the coordinates were stored every 20 ps. The data from the MD simulation trajectories were post-processed using the Schrodinger and VMD scripts (71). Various measurements were obtained to analyze the protein’s structural behavior from the quantitative analysis, including RMSD, RMSF, and MM-GBSA. These measurements provided information about the backbone, side chains, and protein-protein contacts (66, 67, 72–74).

### Binding free energy calculations

The MM-GBSA method was employed for the binding free energy calculations using the PRIME module (75). To determine the average binding energy, the snapshots were extracted from the last stable trajectory for analysis. The binding energy (ΔGbind) was computed using the following equation: ΔGbind = ΔE_MM + ΔG_solv + ΔG_SA. Here, ΔE_MM represents the difference in minimized energies between the protein-protein complex, ΔG_solv corresponds to the difference in GBSA solvation energy between the complexes and the sum of the solvation energies of the CHIKV E1-E2 dimer and AGPAT1, and ΔG_SA denotes the difference in surface area energy between the complex and the sum of the surface area energies of the E1-E2 dimer and AGPAT1 (71). The MM-GBSA approach, together with per-residue energy breakdown analysis, allowed us to determine the energy contributions of individual amino acids to identify critical residues at the interface and to reveal primary residue interactions within the complex by decomposing binding free energy (76).

### Prediction of the critical mutations

The goal of the mutational study was to understand the effect of certain point mutations on the interaction of the CHIKV E1-E2 dimer with AGPAT1. The residue scanning was carried out by BioLuminate (Schrodinger) (77) using the biologics residue scanning panel, side-chain prediction, and backbone minimization as the refinement options, and a cutoff distance of 0.0. BioLuminate calculated the net change in protein stability due to the mutation using the prime energy function, which included an implicit solvent term. The affinity change was calculated using Steepest (Schrodinger). Stability is defined as the free energy difference between the mutant and natural states caused by a single point mutation. BioLuminate (Schrodinger) (77) was used to calculate the energy using the MM-GBSA method, with force field OPLS4 and solvent model VSGB (74).

### Site-directed mutagenesis

The site-directed mutagenesis was done by PCR using the overlapping primers on the plasmids AGPAT1-HA and CHIKV E1-FLAG. Overlapping primers were designed according to the required changes in the coding region. The PCR was done by Phusion polymerase (NEB, M0530S) using 5× HF buffer; the primers designed for the sites are mentioned in Table S1. The PCR product was treated with DpnI (NEB, R0176S) to digest the methylated plasmid (the template), and the resultant product was PCR purified by a PCR purification kit (Qiagen, 28506). The purified DNA was used to transform *E. coli* DH5α cells. The bacteria were grown for 16 h at 37°C, and the plasmid DNA was obtained. Sanger sequencing of the plasmid DNA was used to verify the desired mutation.

### Proximity ligation assay

The Duolink *in situ* Red PLA kit (Sigma Aldrich; DUO92101-1KT) was used following its recommended protocol. Briefly, Huh7 cells were co-transfected with the plasmids or transfected with a plasmid and infected with CHIKV. The cells were washed, fixed, permeabilized, blocked, and incubated with the primary antibodies for 2 h at room temperature. The primary antibodies used were mouse anti-AGPAT1 antibody and rabbit anti-FLAG or rabbit anti-CHIKV E1 antibodies. The rabbit plus and mouse minus probes were used for the rolling circle amplification. The coverslips were mounted in Duolink *in situ* mounting medium with DAPI and imaged under a confocal microscope. ImageJ software quantified the acquired images by measuring the PLA signals per cell. The figures show representative western blot images from multiple experiments.

### Statistical analysis

The statistical analysis on data presented as mean ± SD was done using the Student’s *t*-test with Welch’s correction, unless indicated otherwise. The *P*-values are indicated by stars as *P < 0.05, **P < 0.01, ***P < 0.001, or ns = not significant.

## Data Availability

All data related to this work are included in the article.

## SUPPLEMENTAL MATERIAL

Supplemental material jvi.01733-25-s0001.pdf Figures S1 to S11; Tables S1 to S3.

ASM does not own the copyrights to Supplemental Material that may be linked to, or accessed through, an article. The authors have granted ASM a non-exclusive, world-wide license to publish the Supplemental Material files. Please contact the corresponding author directly for reuse.

## References

[1] Weaver SC, Chen R, Diallo M. 2020. Chikungunya virus: role of vectors in emergence from enzootic cycles. Annu Rev Entomol 65:313–332. doi:10.1146/annurev-ento-011019-025207 31594410

[2] Azar SR, Campos RK, Bergren NA, Camargos VN, Rossi SL. 2020. Epidemic alphaviruses: ecology, emergence and outbreaks. Microorganisms 8:1–35. doi:10.3390/microorganisms8081167 PMC7464724 32752150

[3] de Lima Cavalcanti TYV, Pereira MR, de Paula SO, Franca RF de O. 2022. A review on chikungunya virus epidemiology, pathogenesis and current vaccine development. Viruses 14:969. doi:10.3390/v14050969 35632709 PMC9147731

[4] Ly H. 2024. Ixchiq (VLA1553): the first FDA-approved vaccine to prevent disease caused by chikungunya virus infection. Virulence 15:2301573. doi:10.1080/21505594.2023.2301573 38217381 PMC10793683

[5] Cherian N, Bettis A, Deol A, Kumar A, Di Fabio JL, Chaudhari A, Yimer S, Fahim R, Endy T. 2023. Strategic considerations on developing a CHIKV vaccine and ensuring equitable access for countries in need. NPJ Vaccines 8:123. doi:10.1038/s41541-023-00722-x 37596253 PMC10439111

[6] Jose J, Snyder JE, Kuhn RJ. 2009. A structural and functional perspective of alphavirus replication and assembly. Future Microbiol 4:837–856. doi:10.2217/fmb.09.59 19722838 PMC2762864

[7] Kielian M, Rey FA. 2006. Virus membrane-fusion proteins: more than one way to make a hairpin. Nat Rev Microbiol 4:67–76. doi:10.1038/nrmicro1326 16357862 PMC7097298

[8] Bréhin AC, Rubrecht L, Navarro-Sanchez ME, Maréchal V, Frenkiel MP, Lapalud P, Laune D, Sall AA, Desprès P. 2008. Production and characterization of mouse monoclonal antibodies reactive to chikungunya envelope E2 glycoprotein. Virology (Auckl) 371:185–195. doi:10.1016/j.virol.2007.09.028 17949772

[9] Basore K, Kim AS, Nelson CA, Zhang R, Smith BK, Uranga C, Vang L, Cheng M, Gross ML, Smith J, Diamond MS, Fremont DH. 2019. Cryo-EM structure of chikungunya virus in complex with the Mxra8 receptor. Cell 177:1725–1737. doi:10.1016/j.cell.2019.04.006 31080061 PMC7227486

[10] Song H, Zhao Z, Chai Y, Jin X, Li C, Yuan F, Liu S, Gao Z, Wang H, Song J, Vazquez L, Zhang Y, Tan S, Morel CM, Yan J, Shi Y, Qi J, Gao F, Gao GF. 2019. Molecular basis of arthritogenic alphavirus receptor MXRA8 binding to chikungunya virus envelope protein. Cell 177:1714–1724. doi:10.1016/j.cell.2019.04.008 31080063

[11] Zimmerman O, Zimmerman MI, Raju S, Nelson CA, Errico JM, Madden EA, Holmes AC, Hassan AO, VanBlargan LA, Kim AS, Adams LJ, Basore K, Whitener BM, Palakurty S, Davis-Adams HG, Sun C, Gilliland T, Earnest JT, Ma H, Ebel GD, Zmasek C, Scheuermann RH, Klimstra WB, Fremont DH, Diamond MS. 2023. Vertebrate-class-specific binding modes of the alphavirus receptor MXRA8. Cell 186:4818–4833. doi:10.1016/j.cell.2023.09.007 37804831 PMC10615782

[12] Higgs S, Vanlandingham D. 2015. Chikungunya virus and its mosquito vectors. Vector Borne Zoonotic Dis 15:231–240. doi:10.1089/vbz.2014.1745 25674945

[13] Lo Presti A, Cella E, Angeletti S, Ciccozzi M. 2016. Molecular epidemiology, evolution and phylogeny of chikungunya virus: an updating review. Infect Genet Evol 41:270–278. doi:10.1016/j.meegid.2016.04.006 27085290

[14] Powers AM, Logue CH. 2007. Changing patterns of chikungunya virus: re-emergence of a zoonotic arbovirus. J Gen Virol 88:2363–2377. doi:10.1099/vir.0.82858-0 17698645

[15] Gardner J, Anraku I, Le TT, Larcher T, Major L, Roques P, Schroder WA, Higgs S, Suhrbier A. 2010. Chikungunya virus arthritis in adult wild-type mice. J Virol 84:8021–8032. doi:10.1128/JVI.02603-09 20519386 PMC2916516

[16] Plante K, Wang E, Partidos CD, Weger J, Gorchakov R, Tsetsarkin K, Borland EM, Powers AM, Seymour R, Stinchcomb DT, Osorio JE, Frolov I, Weaver SC. 2011. Novel chikungunya vaccine candidate with an IRES-based attenuation and host range alteration mechanism. PLoS Pathog 7:e1002142. doi:10.1371/journal.ppat.1002142 21829348 PMC3145802

[17] Coffey LL, Failloux AB, Weaver SC. 2014. Chikungunya virus-vector interactions. Viruses 6:4628–4663. doi:10.3390/v6114628 25421891 PMC4246241

[18] Chhabra M, Mittal V, Bhattacharya D, Rana UVS, Lal S. 2008. Chikungunya fever: a re-emerging viral infection. Indian J Med Microbiol 26:5–12. doi:10.4103/0255-0857.38850 18227590

[19] Sourisseau M, Schilte C, Casartelli N, Trouillet C, Guivel-Benhassine F, Rudnicka D, Sol-Foulon N, Le Roux K, Prevost M-C, Fsihi H, et al.. 2007. Characterization of reemerging chikungunya virus. PLoS Pathog 3:e89. doi:10.1371/journal.ppat.0030089 17604450 PMC1904475

[20] Ozden S, Huerre M, Riviere J-P, Coffey LL, Afonso PV, Mouly V, de Monredon J, Roger J-C, El Amrani M, Yvin J-L, Jaffar M-C, Frenkiel M-P, Sourisseau M, Schwartz O, Butler-Browne G, Desprès P, Gessain A, Ceccaldi P-E. 2007. Human muscle satellite cells as targets of chikungunya virus infection. PLoS One 2:e527. doi:10.1371/journal.pone.0000527 17565380 PMC1885285

[21] Schilte C, Couderc T, Chretien F, Sourisseau M, Gangneux N, Guivel-Benhassine F, Kraxner A, Tschopp J, Higgs S, Michault A, Arenzana-Seisdedos F, Colonna M, Peduto L, Schwartz O, Lecuit M, Albert ML. 2010. Type I IFN controls chikungunya virus via its action on nonhematopoietic cells. J Exp Med 207:429–442. doi:10.1084/jem.20090851 20123960 PMC2822618

[22] Gardner CL, Burke CW, Higgs ST, Klimstra WB, Ryman KD. 2012. Interferon-alpha/beta deficiency greatly exacerbates arthritogenic disease in mice infected with wild-type chikungunya virus but not with the cell culture-adapted live-attenuated 181/25 vaccine candidate. Virology (Auckl) 425:103–112. doi:10.1016/j.virol.2011.12.020 PMC3431213 22305131

[23] Reyes Ballista JM, Miazgowicz KL, Acciani MD, Jimenez AR, Belloli RS, Havranek KE, Brindley MA. 2023. Chikungunya virus entry and infectivity is primarily facilitated through cell line dependent attachment factors in mammalian and mosquito cells. Front Cell Dev Biol 11:1085913. doi:10.3389/fcell.2023.1085913 36743418 PMC9895848

[24] McAllister N, Liu Y, Silva LM, Lentscher AJ, Chai W, Wu N, Griswold KA, Raghunathan K, Vang L, Alexander J, Warfield KL, Diamond MS, Feizi T, Silva LA, Dermody TS. 2020. Chikungunya virus strains from each genetic clade bind sulfated glycosaminoglycans as attachment factors. J Virol 94:e01500-20. doi:10.1128/JVI.01500-20 32999033 PMC7925169

[25] Weber C, Berberich E, von Rhein C, Henß L, Hildt E, Schnierle BS. 2017. Identification of functional determinants in the chikungunya virus E2 protein. PLoS Negl Trop Dis 11:e0005318. doi:10.1371/journal.pntd.0005318 28114368 PMC5289616

[26] Zimmerman O, Holmes AC, Kafai NM, Adams LJ, Diamond MS. 2023. Entry receptors - the gateway to alphavirus infection. J Clin Invest 133:e165307. doi:10.1172/JCI165307 36647825 PMC9843064

[27] Zhang R, Kim AS, Fox JM, Nair S, Basore K, Klimstra WB, Rimkunas R, Fong RH, Lin H, Poddar S, Crowe JE, Doranz BJ, Fremont DH, Diamond MS. 2018. Mxra8 is a receptor for multiple arthritogenic alphaviruses. Nature 557:570–574. doi:10.1038/s41586-018-0121-3 29769725 PMC5970976

[28] Wintachai P, Wikan N, Kuadkitkan A, Jaimipuk T, Ubol S, Pulmanausahakul R, Auewarakul P, Kasinrerk W, Weng WY, Panyasrivanit M, Paemanee A, Kittisenachai S, Roytrakul S, Smith DR. 2012. Identification of prohibitin as a chikungunya virus receptor protein. J Med Virol 84:1757–1770. doi:10.1002/jmv.23403 22997079

[29] De Caluwé L, Coppens S, Vereecken K, Daled S, Dhaenens M, Van Ostade X, Deforce D, Ariën KK, Bartholomeeusen K. 2021. The CD147 protein complex is involved in entry of chikungunya virus and related alphaviruses in human cells. Front Microbiol 12:615165. doi:10.3389/fmicb.2021.615165 33717005 PMC7946996

[30] Kirui J, Abidine Y, Lenman A, Islam K, Gwon YD, Lasswitz L, Evander M, Bally M, Gerold G. 2021. The phosphatidylserine receptor TIM-1 enhances authentic chikungunya virus cell entry. Cells 10:1828. doi:10.3390/cells10071828 34359995 PMC8303237

[31] Acosta MP, Geoghegan EM, Lepenies B, Ruzal S, Kielian M, Martinez MG. 2019. Surface (S) layer proteins of Lactobacillus acidophilus block virus infection via DC-SIGN interaction. Front Microbiol 10:810. doi:10.3389/fmicb.2019.00810 31040840 PMC6477042

[32] Agarwal AK, Sukumaran S, Cortés VA, Tunison K, Mizrachi D, Sankella S, Gerard RD, Horton JD, Garg A. 2011. Human 1-acylglycerol-3-phosphate O-acyltransferase isoforms 1 and 2: biochemical characterization and inability to rescue hepatic steatosis in Agpat2(-/-) gene lipodystrophic mice. J Biol Chem 286:37676–37691. doi:10.1074/jbc.M111.250449 21873652 PMC3199511

[33] Aguado B, Campbell RD. 1998. Characterization of a human lysophosphatidic acid acyltransferase that is encoded by a gene located in the class III region of the human major histocompatibility complex. J Biol Chem 273:4096–4105. doi:10.1074/jbc.273.7.4096 9461603

[34] Yap ML, Klose T, Urakami A, Hasan SS, Akahata W, Rossmann MG. 2017. Structural studies of chikungunya virus maturation. Proc Natl Acad Sci USA 114:13703–13707. doi:10.1073/pnas.1713166114 29203665 PMC5748190

[35] Bautista DE, Carr JF, Mitchell AM. 2021. Suppressor mutants: history and today’s applications. EcoSal Plus 9:eESP00372020. doi:10.1128/ecosalplus.ESP-0037-2020 34910591 PMC9008745

[36] Lentscher AJ, McCarthy MK, May NA, Davenport BJ, Montgomery SA, Raghunathan K, McAllister N, Silva LA, Morrison TE, Dermody TS. 2020. Chikungunya virus replication in skeletal muscle cells is required for disease development. J Clin Invest 130:1466–1478. doi:10.1172/JCI129893 31794434 PMC7269570

[37] Rohatgi A, Corbo JC, Monte K, Higgs S, Vanlandingham DL, Kardon G, Lenschow DJ. 2014. Infection of myofibers contributes to increased pathogenicity during infection with an epidemic strain of chikungunya virus. J Virol 88:2414–2425. doi:10.1128/JVI.02716-13 24335291 PMC3958092

[38] Hawman DW, Carpentier KS, Fox JM, May NA, Sanders W, Montgomery SA, Moorman NJ, Diamond MS, Morrison TE. 2017. Mutations in the E2 glycoprotein and the 3′ untranslated region enhance chikungunya virus virulence in mice. J Virol 91:e00816-17. doi:10.1128/JVI.00816-17 28747508 PMC5625495

[39] Nair S, Poddar S, Shimak RM, Diamond MS. 2017. Interferon regulatory factor 1 protects against chikungunya virus-induced immunopathology by restricting infection in muscle cells. J Virol 91:e01419-17. doi:10.1128/JVI.01419-17 PMC5660487 28835505

[40] Couderc T, Chrétien F, Schilte C, Disson O, Brigitte M, Guivel-Benhassine F, Touret Y, Barau G, Cayet N, Schuffenecker I, Desprès P, Arenzana-Seisdedos F, Michault A, Albert ML, Lecuit M. 2008. A mouse model for chikungunya: young age and inefficient type-I interferon signaling are risk factors for severe disease. PLoS Pathog 4:e29. doi:10.1371/journal.ppat.0040029 18282093 PMC2242832

[41] Feng F, Bouma EM, Hu G, Zhu Y, Yu Y, Smit JM, Diamond MS, Zhang R. 2023. Colocalization of chikungunya virus with its receptor MXRA8 during cell attachment, internalization, and membrane fusion. J Virol 97:e0155722. doi:10.1128/jvi.01557-22 37133449 PMC10231136

[42] Zhang R, Earnest JT, Kim AS, Winkler ES, Desai P, Adams LJ, Hu G, Bullock C, Gold B, Cherry S, Diamond MS. 2019. Expression of the Mxra8 receptor promotes alphavirus infection and pathogenesis in mice and Drosophila. Cell Rep 28:2647–2658. doi:10.1016/j.celrep.2019.07.105 31484075 PMC6745702

[43] Kim AS, Zimmerman O, Fox JM, Nelson CA, Basore K, Zhang R, Durnell L, Desai C, Bullock C, Deem SL, Oppenheimer J, Shapiro B, Wang T, Cherry S, Coyne CB, Handley SA, Landis MJ, Fremont DH, Diamond MS. 2020. An evolutionary insertion in the mxra8 receptor-binding site confers resistance to alphavirus infection and pathogenesis. Cell Host & Microbe 27:428–440. doi:10.1016/j.chom.2020.01.008 32075743 PMC7163869

[44] Subauste AR, Elliott B, Das AK, Burant CF. 2010. A role for 1-acylglycerol-3-phosphate-O-acyltransferase-1 in myoblast differentiation. Differentiation 80:140–146. doi:10.1016/j.diff.2010.05.006 20561744 PMC3449212

[45] Agarwal AK, Tunison K, Dalal JS, Nagamma SS, Hamra FK, Sankella S, Shao X, Auchus RJ, Garg A. 2017. Metabolic, reproductive, and neurologic abnormalities in agpat1-null mice. Endocrinology 158:3954–3973. doi:10.1210/en.2017-00511 28973305 PMC5695831

[46] Tabata K, Prasad V, Paul D, Lee J-Y, Pham M-T, Twu W-I, Neufeldt CJ, Cortese M, Cerikan B, Stahl Y, et al.. 2021. Convergent use of phosphatidic acid for hepatitis C virus and SARS-CoV-2 replication organelle formation. Nat Commun 12:7276. doi:10.1038/s41467-021-27511-1 34907161 PMC8671429

[47] Milewska A, Nowak P, Owczarek K, Szczepanski A, Zarebski M, Hoang A, Berniak K, Wojarski J, Zeglen S, Baster Z, Rajfur Z, Pyrc K. 2018. Entry of human coronavirus NL63 into the cell. J Virol 92. doi:10.1128/JVI.01933-17 PMC5774871 29142129

[48] Liebl D, Difato F, Horníková L, Mannová P, Stokrová J, Forstová J. 2006. Mouse polyomavirus enters early endosomes, requires their acidic pH for productive infection, and meets transferrin cargo in Rab11-positive endosomes. J Virol 80:4610–4622. doi:10.1128/JVI.80.9.4610-4622.2006 16611921 PMC1472029

[49] Katsarou K, Lavdas AΑ, Tsitoura P, Serti E, Markoulatos P, Mavromara P, Georgopoulou U. 2010. Endocytosis of hepatitis C virus non-enveloped capsid-like particles induces MAPK–ERK1/2 signaling events. Cell Mol Life Sci 67:2491–2506. doi:10.1007/s00018-010-0351-5 20358251 PMC11115770

[50] Mukhopadhyay S, Zhang W, Gabler S, Chipman PR, Strauss EG, Strauss JH, Baker TS, Kuhn RJ, Rossmann MG. 2006. Mapping the structure and function of the E1 and E2 glycoproteins in alphaviruses. Structure 14:63–73. doi:10.1016/j.str.2005.07.025 16407066 PMC2757649

[51] Yamashita A, Hayashi Y, Matsumoto N, Nemoto-Sasaki Y, Oka S, Tanikawa T, Sugiura T. 2014. Glycerophosphate/acylglycerophosphate acyltransferases. Biology (Basel) 3:801–830. doi:10.3390/biology3040801 25415055 PMC4280512

[52] Teymournejad O, Mobarez AM, Hassan ZM, Moazzeni SM, Yakhchali B, Eskandari V. 2012. In silico prediction of exposure amino acid sequences of outer inflammatory protein A of Helicobacter pylori for surface display on Eschierchia coli. Indian J Hum Genet 18:83–86. doi:10.4103/0971-6866.96659 22754227 PMC3385185

[53] Brosig B, Langosch D. 1998. The dimerization motif of the glycophorin A transmembrane segment in membranes: importance of glycine residues. Protein Sci 7:1052–1056. doi:10.1002/pro.5560070423 9568912 PMC2143994

[54] Lemmon MA, Flanagan JM, Hunt JF, Adair BD, Bormann BJ, Dempsey CE, Engelman DM. 1992. Glycophorin A dimerization is driven by specific interactions between transmembrane alpha-helices. J Biol Chem 267:7683–7689. doi:10.1016/s0021-9258(18)42569-0 1560003

[55] Konovalova A, Perlman DH, Cowles CE, Silhavy TJ. 2014. Transmembrane domain of surface-exposed outer membrane lipoprotein RcsF is threaded through the lumen of β-barrel proteins. Proc Natl Acad Sci USA 111:E4350–E4358. doi:10.1073/pnas.1417138111 25267629 PMC4205638

[56] Voss BJ, Gaddy JA, McDonald WH, Cover TL. 2014. Analysis of surface-exposed outer membrane proteins in Helicobacter pylori. J Bacteriol 196:2455–2471. doi:10.1128/JB.01768-14 24769695 PMC4054173

[57] Minajigi A, Francklyn CS. 2008. RNA-assisted catalysis in a protein enzyme: The 2’-hydroxyl of tRNA(Thr) A76 promotes aminoacylation by threonyl-tRNA synthetase. Proc Natl Acad Sci USA 105:17748–17753. doi:10.1073/pnas.0804247105 18997014 PMC2584683

[58] Bloch S, Tomek MB, Friedrich V, Messner P, Schäffer C. 2019. Nonulosonic acids contribute to the pathogenicity of the oral bacterium Tannerella forsythia. Interface Focus 9:20180064. doi:10.1098/rsfs.2018.0064 30842870 PMC6388019

[59] Herker E. 2024. Lipid droplets in virus replication. FEBS Lett 598:1299–1300. doi:10.1002/1873-3468.14819 38348563 PMC12628354

[60] Ali MM, Mcmillan RP, Fausnacht DW, Kavanaugh JW, Harvey MM, Stevens JR, Wu Y, Mynatt RL, Hulver MW. 2021. Muscle-specific deletion of toll-like receptor 4 impairs metabolic adaptation to wheel running in mice. Med Sci Sports Exerc 53:1161–1169. doi:10.1249/MSS.0000000000002579 33315811

[61] Agarwal AK, Garg A. 2010. Enzymatic activity of the human 1-acylglycerol-3-phosphate-O-acyltransferase isoform 11: upregulated in breast and cervical cancers. J Lipid Res 51:2143–2152. doi:10.1194/jlr.M004762 20363836 PMC2903799

[62] Thakur R, Naik A, Panda A, Raghu P. 2019. Regulation of membrane turnover by phosphatidic acid: cellular functions and disease implications. Front Cell Dev Biol 7:83. doi:10.3389/fcell.2019.00083 31231646 PMC6559011

[63] Antonescu CN, Danuser G, Schmid SL. 2010. Phosphatidic acid plays a regulatory role in clathrin-mediated endocytosis. Mol Biol Cell 21:2944–2952. doi:10.1091/mbc.E10-05-0421 20573978 PMC2921119

[64] Kumar S, Nagesh D, Ramasubbu V, Prabhashankar AB, Sundaresan NR. 2023. Isolation and culture of primary fibroblasts from neonatal murine hearts to study cardiac fibrosis. Bio Protoc 13:e4616. doi:10.21769/BioProtoc.4616 PMC9947550 36845532

[65] Jumper J, Evans R, Pritzel A, Green T, Figurnov M, Ronneberger O, Tunyasuvunakool K, Bates R, Žídek A, Potapenko A, et al.. 2021. Highly accurate protein structure prediction with AlphaFold. Nature 596:583–589. doi:10.1038/s41586-021-03819-2 34265844 PMC8371605

[66] Kumari A, Mittal L, Srivastava M, Asthana S. 2022. Binding mode characterization of 13b in the monomeric and dimeric states of SARS-CoV-2 main protease using molecular dynamics simulations. J Biomol Struct Dyn 40:9287–9305. doi:10.1080/07391102.2021.1927844 34029506

[67] Srivastava M, Mittal L, Kumari A, Asthana S. 2021. Molecular dynamics simulations reveal the interaction fingerprint of remdesivir triphosphate pivotal in allosteric regulation of SARS-CoV-2 RdRp. Front Mol Biosci 8:639614. doi:10.3389/fmolb.2021.639614 34490343 PMC8417884

[68] Roos K, Wu C, Damm W, Reboul M, Stevenson JM, Lu C, Dahlgren MK, Mondal S, Chen W, Wang L, Abel R, Friesner RA, Harder ED. 2019. OPLS3e: extending force field coverage for drug-like small molecules. J Chem Theory Comput 15:1863–1874. doi:10.1021/acs.jctc.8b01026 30768902

[69] Kumari A, Mittal L, Srivastava M, Pathak DP, Asthana S. 2023. Deciphering the structural determinants critical in attaining the FXR partial agonism. J Phys Chem B 127:465–485. doi:10.1021/acs.jpcb.2c06325 36609158

[70] Asthana S, Shukla S, Vargiu AV, Ceccarelli M, Ruggerone P, Paglietti G, Marongiu ME, Blois S, Giliberti G, La Colla P. 2013. Different molecular mechanisms of inhibition of bovine viral diarrhea virus and hepatitis C virus RNA-dependent RNA polymerases by a novel benzimidazole. Biochemistry 52:3752–3764. doi:10.1021/bi400107h 23627712

[71] Mittal L, Kumari A, Suri C, Bhattacharya S, Asthana S. 2020. Insights into structural dynamics of allosteric binding sites in HCV RNA-dependent RNA polymerase. J Biomol Struct Dyn 38:1–14. doi:10.1080/07391102.2019.1614480 31057089

[72] Mittal L, Tonk RK, Awasthi A, Asthana S. 2021. Targeting cryptic-orthosteric site of PD-L1 for inhibitor identification using structure-guided approach. Arch Biochem Biophys 713:109059. doi:10.1016/j.abb.2021.109059 34673001

[73] Kumari A, Mittal L, Srivastava M, Pathak DP, Asthana S. 2021. Conformational characterization of the co-activator binding site revealed the mechanism to achieve the bioactive state of FXR. Front Mol Biosci 8:658312. doi:10.3389/fmolb.2021.658312 34532338 PMC8439381

[74] Srivastava M, Mittal L, Sarmadhikari D, Singh VK, Fais A, Kumar A, Asthana S. 2023. Template entrance channel as possible allosteric inhibition and resistance site for quinolines tricyclic derivatives in RNA dependent RNA polymerase of bovine viral diarrhea virus. Pharmaceuticals (Basel) 16:376. doi:10.3390/ph16030376 36986476 PMC10058290

[75] Mittal L, Kumari A, Srivastava M, Singh M, Asthana S. 2021. Identification of potential molecules against COVID-19 main protease through structure-guided virtual screening approach. J Biomol Struct Dyn 39:3662–3680. doi:10.1080/07391102.2020.1768151 32396769 PMC7256355

[76] Genheden S, Ryde U. 2015. The MM/PBSA and MM/GBSA methods to estimate ligand-binding affinities. Expert Opin Drug Discov 10:449–461. doi:10.1517/17460441.2015.1032936 25835573 PMC4487606

[77] Zhu K, Day T, Warshaviak D, Murrett C, Friesner R, Pearlman D. 2014. Antibody structure determination using a combination of homology modeling, energy-based refinement, and loop prediction. Proteins 82:1646–1655. doi:10.1002/prot.24551 24619874 PMC5282925

