# Supplementary material for: AGPAT1 is a novel Chikungunya virus receptor on human cells
Source: J Virol. 2026 Feb 17;100(3):e01733-25. doi: 10.1128/jvi.01733-25 (PMC13011415; doi:10.1128/jvi.01733-25)
Supplement: Supplemental material — Figures S1 to S11; Tables S1 to S3. [file jvi.01733-25-s0001.pdf]

Figure S1

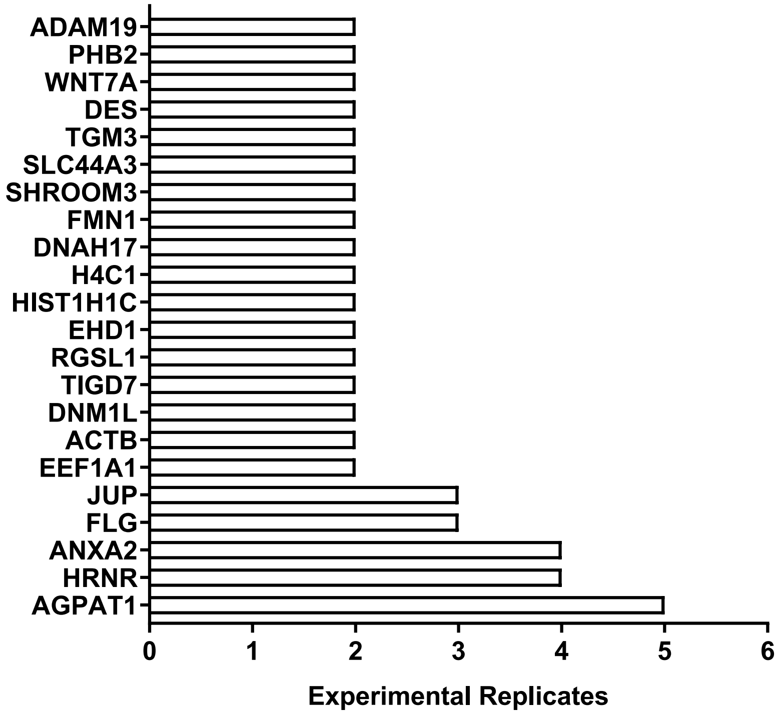

Figure S1. Identification of CHIKV-binding plasma membrane proteins.

Purified CHIKV conjugated with agarose beads was incubated with the plasma membrane proteins from Huh7 cells. The pulled-down proteins were identified by mass spectrometry. The figure shows the proteins identified two or more times from the six independent experiments. The NCBI IDs of the proteins are shown on the left.

Figure S2

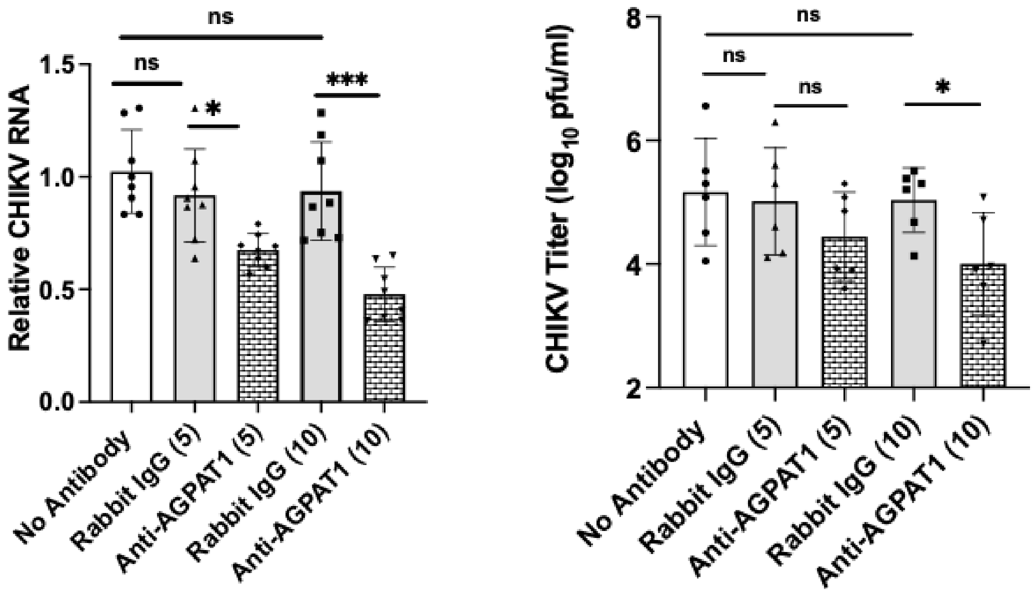

Figure S2. CHIKV replication in the presence of AGPAT1 antibody.

Huh7 cells were incubated with 5 or 10 µg/ml concentration (indicated in the brackets) of rabbit anti-AGPAT1 antibody or rabbit IgG (isotype control) on ice for 30 min, or without antibody, and then incubated with CHIKV (MOI 1) on ice for 1 h. The cells were then washed with ice-cold PBS and incubated at 37 ºC. The culture supernatant and the cells were harvested 6 h pi. The total RNA from the cells was isolated, and relative CHIKV RNA level was determined by qRT-PCR (left panel). The CHIKV RNA level in the no-antibody control was taken as 1. The viral titers in the culture supernatant are presented in the right panel. The data from 3 biological replicates and 2 technical replicates are shown and analysed by One-way Anova.

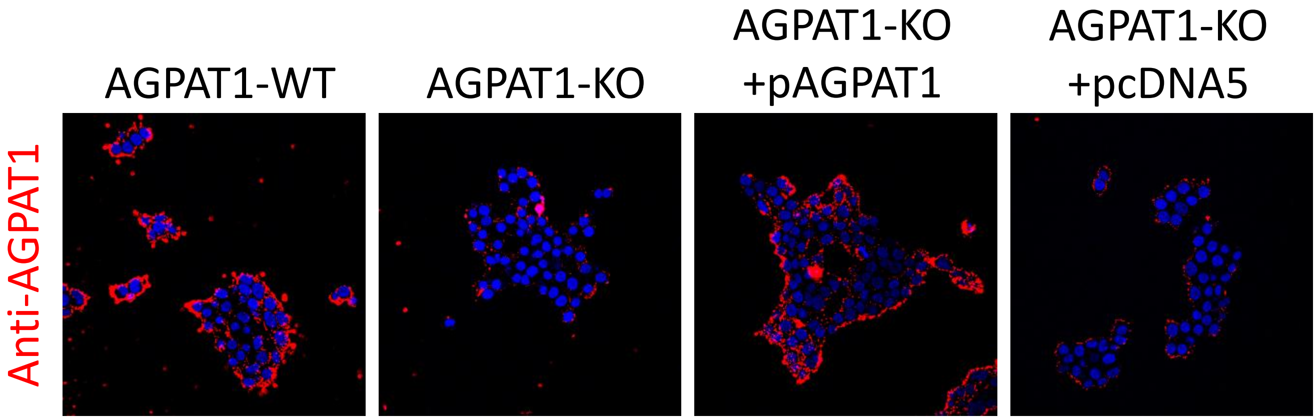

Figure S3. CHIKV binding in AGPAT1 knockout HAP1 cells.

The HAP1 AGPAT1 KO cells transfected with pAGPAT1-HA or pcDNA5 were fixed and incubated with CHIKV (MOI 25) on ice for 30 min. This was followed by staining the cells with CHIKV anti-E2 mouse antibody and Alexa Fluor-647 anti-mouse antibody, and microscopy for MIFD determination.

Figure S4

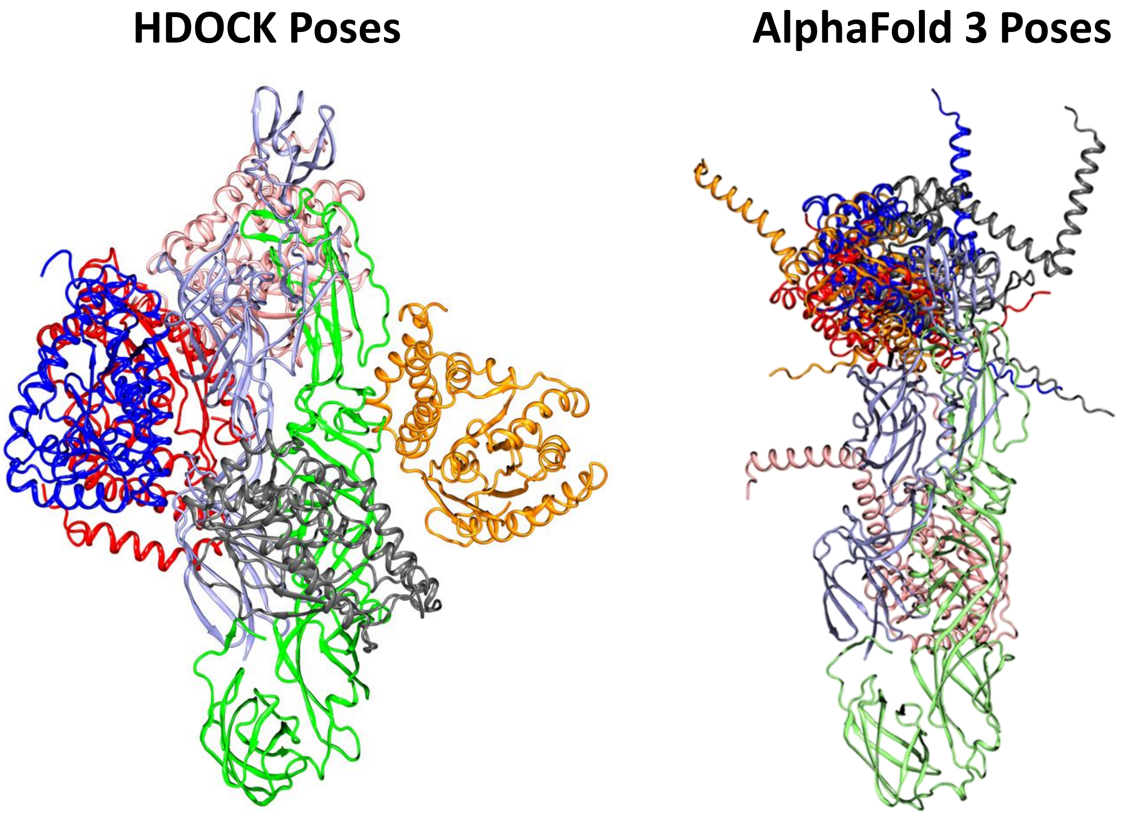

Figure S4: The top poses obtained from the protein-protein docking.

The top 5 poses of AGPAT1 interaction with the CHIKV E1-E2 dimer predicted using the HDOCK and AlphaFold 3 methods are presented. The top poses of AGPAT1 in the complexes (from pose-1 to pose-5) are depicted in grey, orange, pink, blue and red, respectively. The CHIKV E1 in shown in green and E2 in ice blue colour.

Figure S5

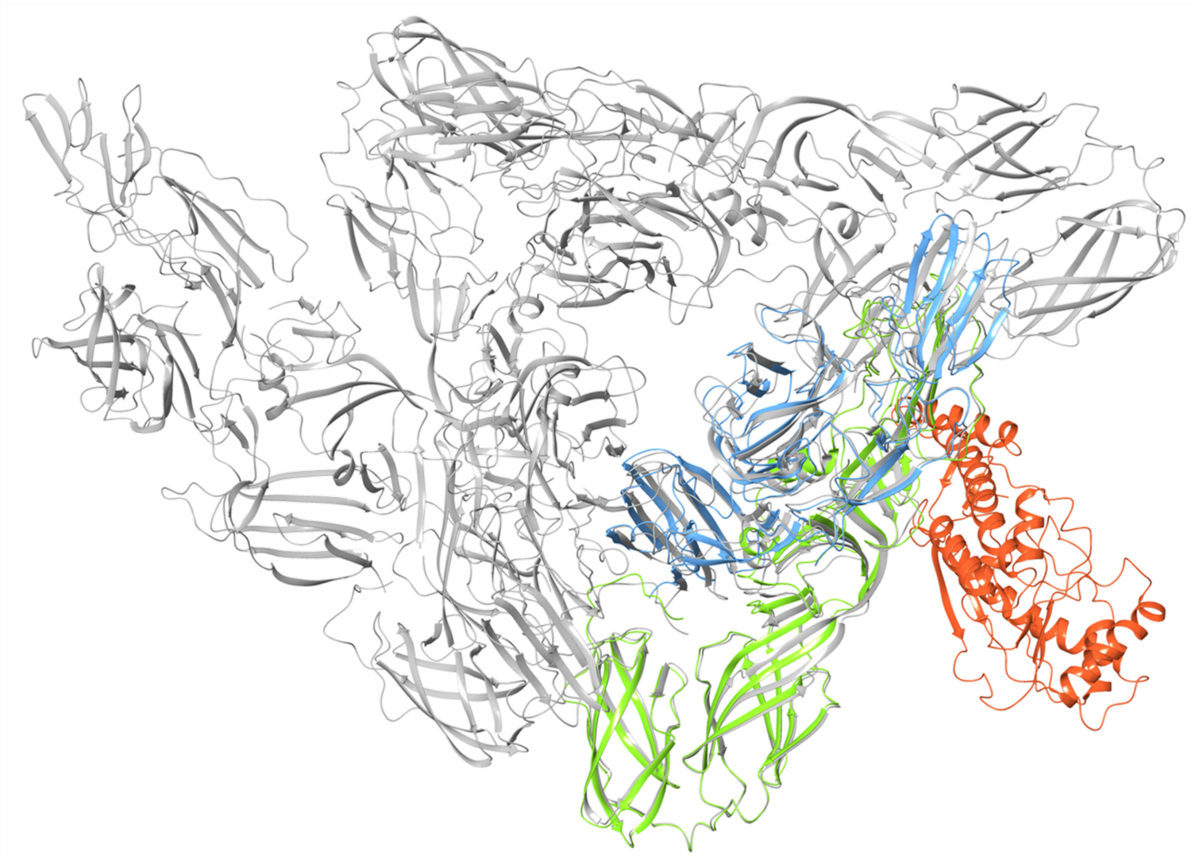

Figure S5. AGPAT1 and CHIKV E1-E2 dimer complex superimposed on a single spike unit of CHIKV envelope.

The predicted complex (pose-2) of E1-E2 with AGPAT1 (E1 is colored green, E2 is colored ice blue, and AGPAT1 is colored orange) shown with full E1-E2 trimeric spike (PDB ID: 2XFB, colored grey).

Figure S6

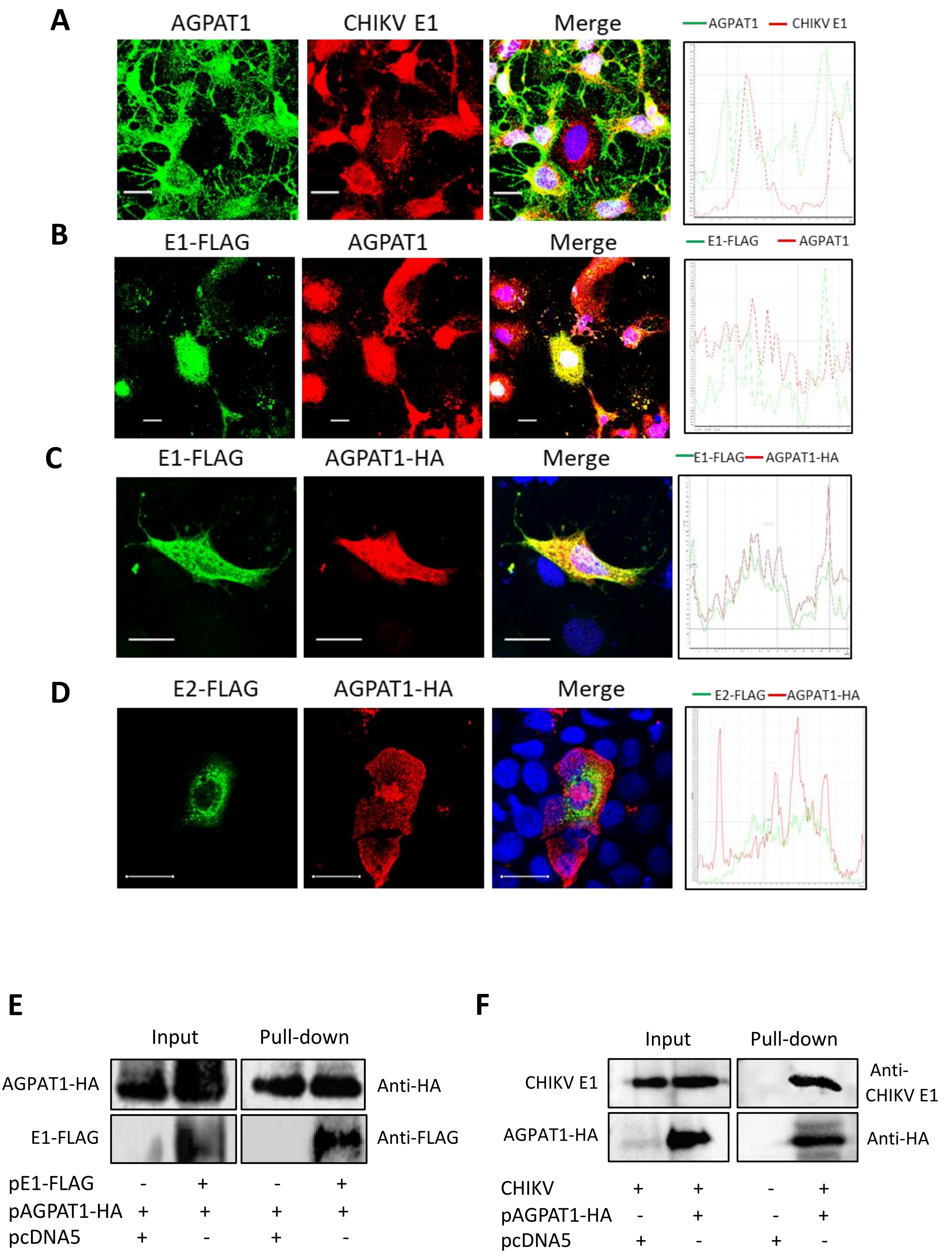
A
AGPAT1
CHIKV E1
Merge
AGPAT1
CHIKV E1
B
E1-FLAG
AGPAT1
Merge
E1-FLAG
AGPAT1
C
E1-FLAG
AGPAT1-HA
Merge
E1-FLAG
AGPAT1-HA
D
E2-FLAG
AGPAT1-HA
Merge
E2-FLAG
AGPAT1-HA
E
Input
Pull-down
AGPAT1-HA
Anti-HA
E1-FLAG
Anti-FLAG
pE1-FLAG - + - +
pAGPAT1-HA + + + +
pcDNA5 + - + -
F
Input
Pull-down
CHIKV E1
Anti-CHIKV E1
AGPAT1-HA
Anti-HA
CHIKV + + - +
pAGPAT1-HA - + - +
pcDNA5 + - + -

Figure S6. CHIKV E1 interacts with AGPAT1 in Huh7 cells.

(A) Huh7 cells were infected with CHIKV (MOI 5), and 24 h later were fixed and permeabilized. The cells were stained with rabbit anti-AGPAT1 polyclonal antibody and mouse CHIKV anti-E1 monoclonal antibody, followed by incubation with anti-mouse Alexa Fluor-647 and anti-rabbit Alexa Fluor-488 antibodies. The nuclei were stained with DAPI, and the cells were imaged (scale bar = 20 µm). The PCC was determined for the colocalization of Alexa Fluor-488 (AGPAT1) and Alexa Fluor-647 (CHIKV E1) dyes. The right panel shows the line plot showing the colocalization of the red (CHIKV E1) and green (AGPAT1) fluorescence signals. (B) Huh7 cells were transfected with the plasmid expressing the tagged protein CHIKV E1-FLAG (pE1-FLAG), and 48 h later were fixed and permeabilized. The cells were stained with rabbit anti-AGPAT1 polyclonal antibody and mouse anti-FLAG monoclonal antibody, followed by incubation with anti-mouse Alexa Fluor-488 and anti-rabbit Alexa Fluot-568 antibodies. The nuclei were stained with DAPI, and the cells were imaged (scale bar = 20 µm). The PCC was determined for the colocalization of the Alexa Fluor-568 (AGPAT1) and Alexa Fluor-488 (CHIKV E1) dyes. The right panel shows the line plot showing the colocalization of the green (CHIKV E1) and red (AGPAT1) fluorescence signals. (C) Huh7 cells were transfected with the plasmids pE1-FLAG and pAGPAT1-HA. The cells were fixed 48 h later, permeabilized, and stained with rabbit anti-HA and mouse anti-FLAG antibodies. This was followed by incubation with anti-mouse Alexa Fluor-488 and anti-rabbit Alexa-568 antibodies. The nuclei were stained with DAPI, and the cells were imaged (scale bar = 20 µm). The PCC was determined for the colocalization of the Alexa Fluor-568 (AGPAT1) and Alexa Fluor-488 (CHIKV E1) dyes. The right panel shows the line plot showing the colocalization of the green (CHIKV E1) and red (AGPAT1) fluorescence signals. (D) Huh7 cells were co-transfected with plasmids expressing AGPAT1-HA and CHIKV E2-FLAG. The cells were fixed, permeabilized and stained with rabbit anti-HA and mouse anti-FLAG antibodies. The cells were further incubated with anti-mouse Alexa Flour-488 and anti-rabbit Alexa-568 antibodies. The nuclei were stained with DAPI, and the cells were imaged (scale bar = 20 µm). The PCC was determined for the colocalization of the Alexa Flour-488 (CHIKV E2) and Alexa Fluor-568 (AGPAT1) dyes. The right panel has the line plot for the two dyes. (E) Huh7 cells were transfected with the plasmids pAGPAT1, pE1-FLAG, or pcDNA5 as indicated. The cell lysates were prepared 48 h later and immunoprecipitated with anti-HA antibody. The pull-down and input samples were Western blotted with anti-HA antibody for detecting the AGPAT1-HA protein or anti-FLAG antibody for detecting the CHIKV E1-FLAG protein. (F) Huh7 cells were transfected with pAGPAT1-HA or pcDNA5, and 48 h later infected with CHIKV (MOI 3). The cell lysates were prepared 24 h pi and immunoprecipitated with anti-HA antibody. The pull-down and input samples were Western blotted with anti-HA antibody to detect the AGPAT1-HA protein and anti-E1 antibody to detect the CHIKV E1 protein.

Figure S7

A

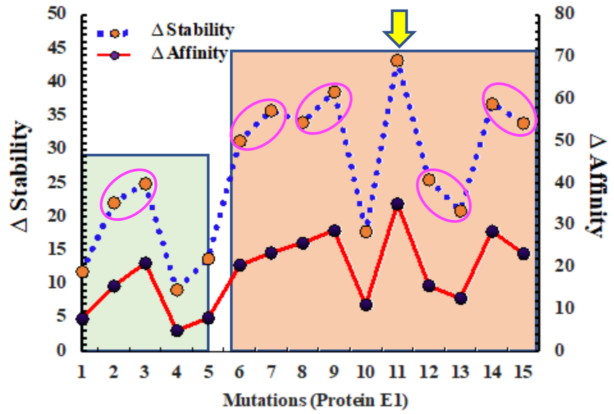

B

Mutation 1	PRO 74	ALA		
Mutation 2	PHE 192	ALA		
Mutation 3	ARG 206	ALA		
Mutation 4	ASN 216	ALA		
Mutation 5	GLN 218	ALA		
Mutation 6	PRO 74	ALA	PHE 192	ALA
Mutation 7	PRO 74	ALA	ARG 206	ALA
Mutation 8	PRO 74	ALA	ASN 216	ALA
Mutation 9	PRO 74	ALA	GLN 218	ALA
Mutation 10	PHE 192	ALA	ARG 206	ALA
Mutation 11	PHE 192	ALA	ASN 216	ALA
Mutation 12	PHE 192	ALA	GLN 218	ALA
Mutation 13	ARG 206	ALA	ASN 216	ALA
Mutation 14	ARG 206	ALA	GLN 218	ALA
Mutation 15	ASN 216	ALA	GLN 218	ALA

Protein E1 mutated residues

C

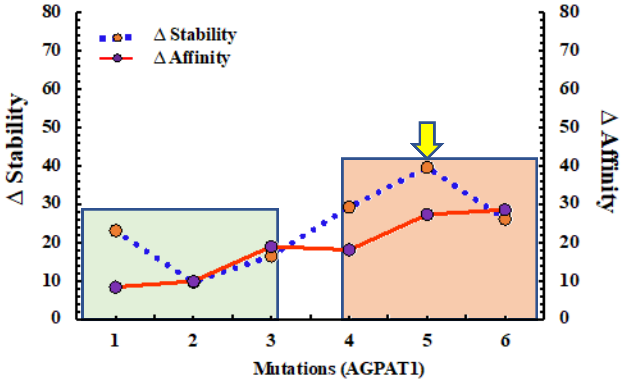

D

Mutation 1	LEU 19	ALA
Mutation 2	PHE 20	ALA
Mutation 3	TYR 78	ALA
Mutation 4	LEU 19<br>PHE 20	ALA
Mutation 5	LEU 19<br>TYR 78	ALA
Mutation 6	PHE 20<br>TYR 78	ALA

AGPAT1 Mutated residues

Figure S7. ***In-silico*** **alanine scanning for identification of the critical residues.**

The residue scanning was carried out by BioLuminate (Schrodinger) as described in the methods. Based on interaction fingerprinting, the residues target for mutation to alanine are shown for E1 (A and B panels) and for AGPAT1 (C and D panels). The effect of the mutation on the complex affinity and stability is depicted in the plots (A and C panels) and selected residues are shown (B and D panels). All energies of stability (in dotted blue line) and affinity (red line) were measured in kcal/mol. The single mutations are depicted in the olive-green box and double mutations are depicted in the peach box. The most stable double mutations *i.e.* mutation 11 of E1 (A panel) and mutation 5 of AGPAT1 (panel C) are highlighted by yellow arrow.

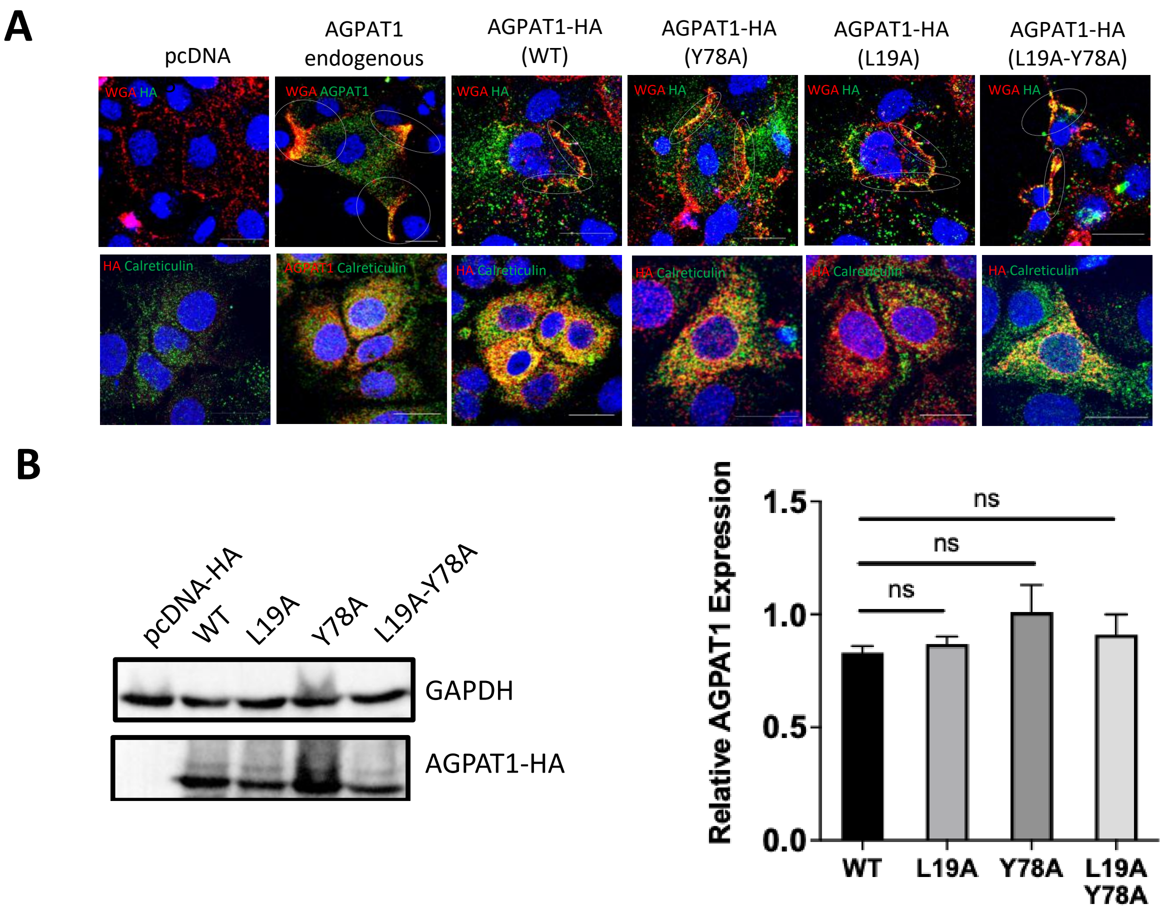

Figure S8. Expression of AGPAT1 mutants in Huh cells.

(A) Huh cells were transfected with plasmids expressing the wild type and mutant AGPAT1 protein tagged with HA. The cells were stained 48 h later with rabbit anti-HA and Alexa 488 anti-rabbit antibodies to localize the ectopically-expressed protein. The endogenous AGPAT1 was stained with rabbit anti-AGPAT1 and Alexa 488 anti-rabbit antibodies. Wheat Germ Agglutinin (WGA)-Alexa 647 conjugate (W32466; Invitrogen) was used to localize the plasma membrane as shown in the upper panel. The lower panel shows the co-localization of AGPAT1 with Calreticulin an endoplasmic reticulum marker, showing the conventionally reported location of AGPAT1 in the endoplasmic reticulum in Huh7 cells. The cells were stained with mouse anti-HA or anti-AGPAT1 and Alexa 488 anti-mouse antibodies. Calreticulin was stained with rabbit anti-calreticulin (12238; Cell Signalling Technologies), and Alexa 568 anti-rabbit antibodies. (B) Huh cells were transfected with plasmids expressing the wild type and mutant AGPAT1 protein tagged with HA. The cells were lysed 48 h later and western blotted with rabbit anti-HA antibody (left panel). GAPDH stained with rabbit anti-GAPDH antibody was used as the internal control. The intensity of the AGPAT1 bands was normalized to GAPDH and the the relative expression levels of AGPAT1 are presented (right panel). The statistical analysis was done by the ordinary one-way ANOVA with Dunnett's multiple comparisons test considering the AGPAT1-WT expression levels as the control.

Figure S9

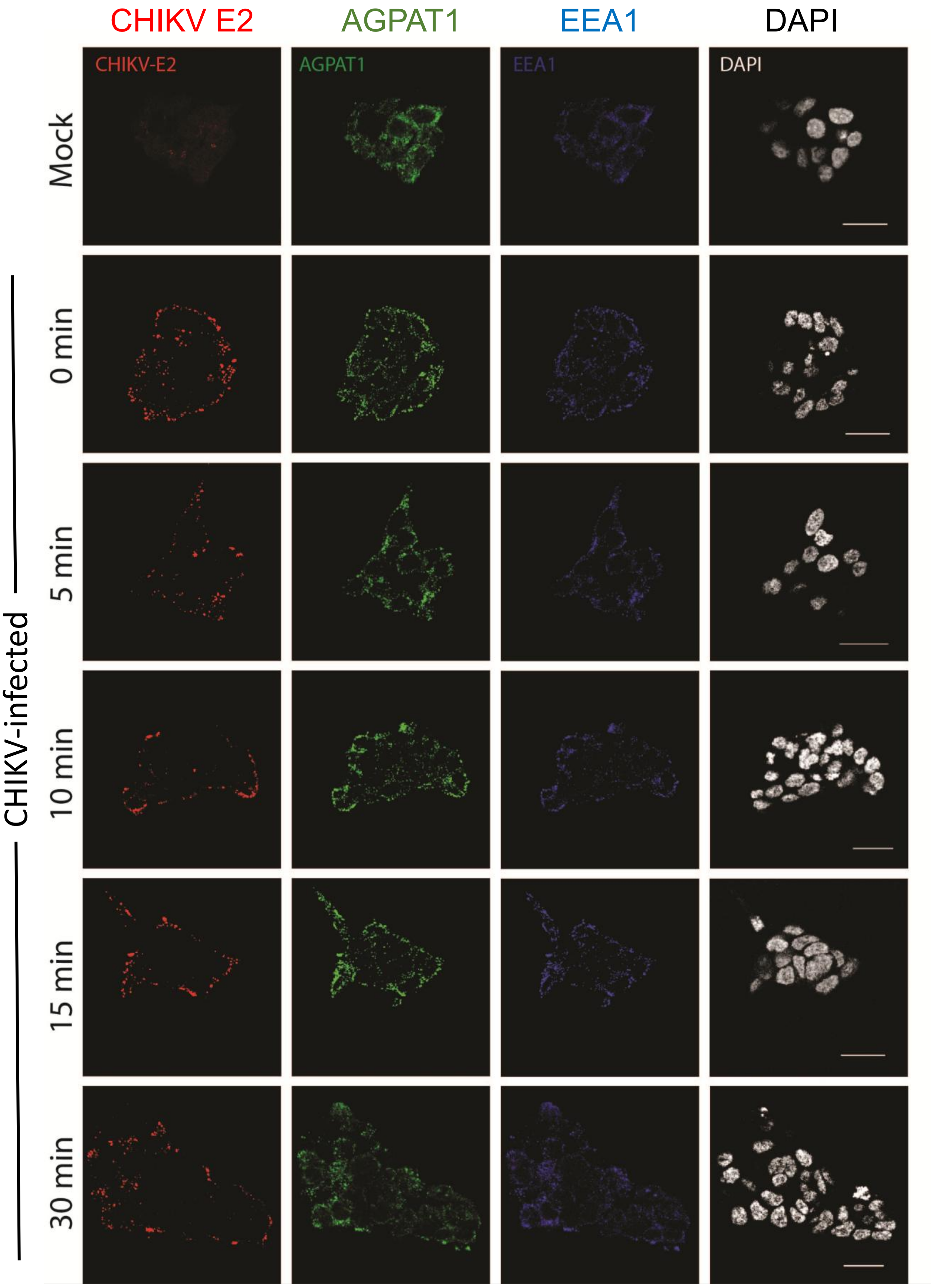

CHIKV E2
AGPAT1
EEA1
DAPI
CHIKV-E2
AGPAT1
EEA1
DAPI
Mock
0 min
5 min
10 min
15 min
30 min
CHIKV-infected

Figure S9. CHIKV and AGPAT1 co-localize in the early endosomes.

Wild-type HAP1 cells were infected with CHIKV (50 MOI). The cells were fixed at different times pi with 4% para-formaldehyde and permeabilized by 0.3% Tween-20 before staining with anti-CHIKV E2 antibody for CHIKV, anti-AGPAT1 antibody for AGPAT1, and anti-EEA1 antibody for early endosome protein EEA1, followed by imaging. CHIKV-E2 was tagged with Alexa 647 (anti-mouse), AGPAT1 with Alexa 488 (anti-rabbit), and EEA1 with Alexa 568 (anti-goat). Scale = 20 μm.

Figure S10

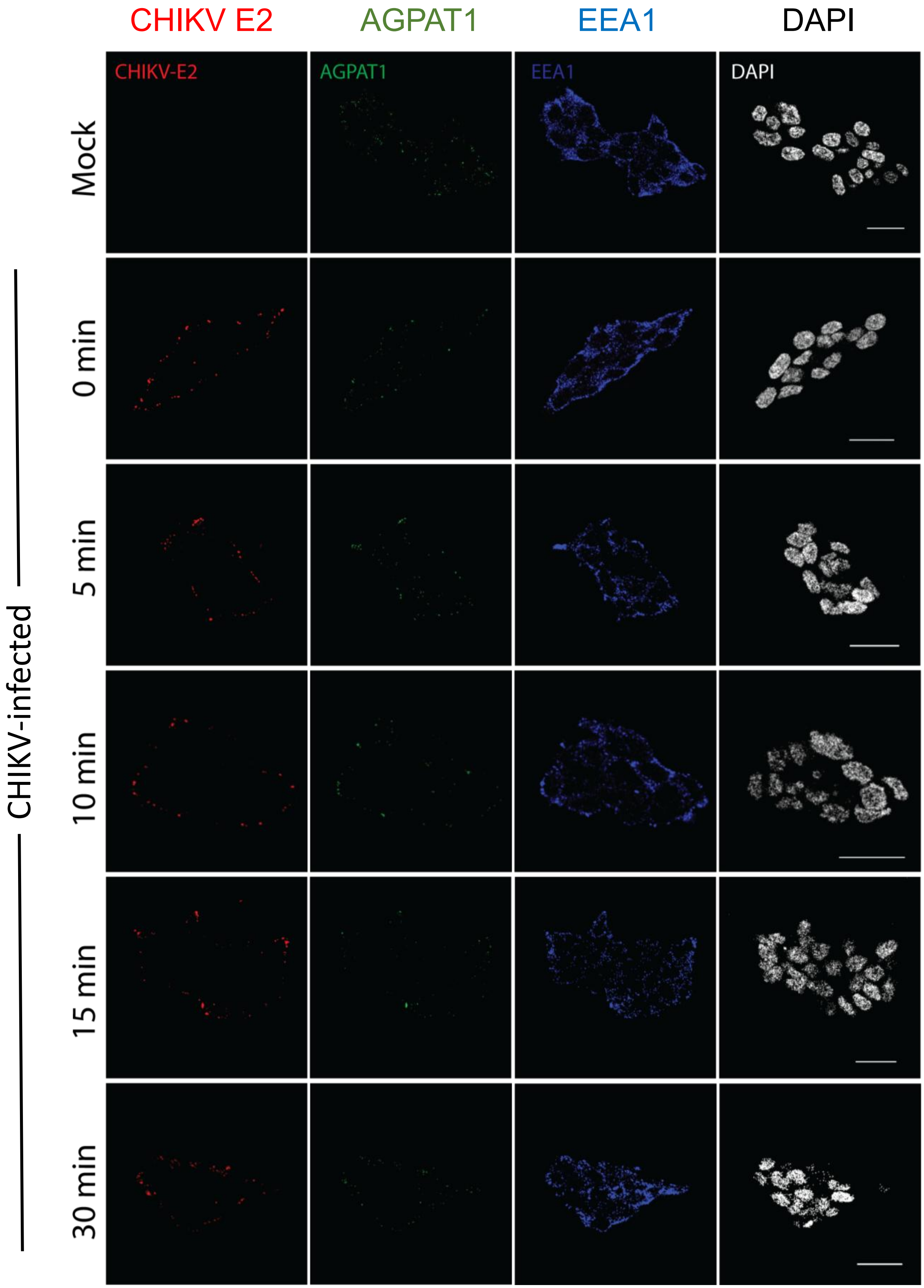
CHIKV E2
AGPAT1
EEA1
DAPI
CHIKV-E2
AGPAT1
EEA1
DAPI
Mock
0 min
5 min
10 min
15 min
30 min
CHIKV-infected

Figure S10. CHIKV and AGPAT1 co-localize in the early endosomes.

AGPAT1-KO HAP1 cells were infected with CHIKV (50 MOI). The cells were fixed at different times pi with 4% para-formaldehyde and permeabilized by 0.3% Tween-20 before staining with anti-CHIKV E2 antibody for CHIKV, anti-AGPAT1 antibody for AGPAT1, and anti-EEA1 antibody for early endosome protein EEA1, followed by imaging. CHIKV-E2 was tagged with Alexa 647 (anti-mouse), AGPAT1 with Alexa 488 (anti-rabbit), and EEA1 with Alexa 568 (anti-goat). Scale = 20 µm.

Figure S11

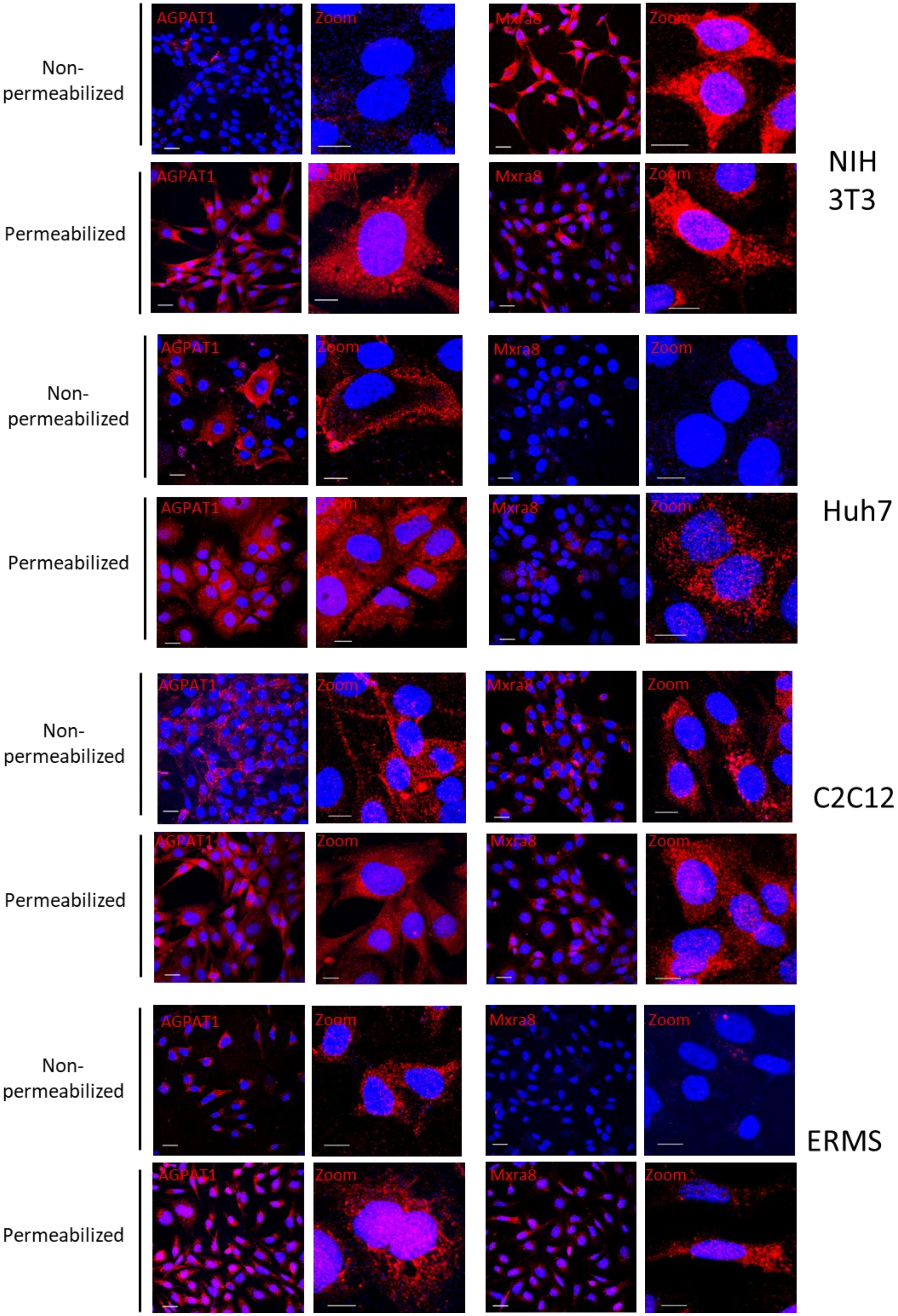
Non-permeabilized
Permeabilized
Non-permeabilized
Permeabilized
Non-permeabilized
Permeabilized
Non-permeabilized
Permeabilized
AGPAT1
Zoom
Mxra8
Zoom
NIH 3T3
Huh7
C2C12
ERMS

Figure S11. Localization of AGPAT1 and MXRA8 on different cell lines.

The cells were permeabilized with 0.3% Tween-20 or were not permeabilized, and incubated with polyclonal rabbit AGPAT1 or polyclonal rabbit MXRA8 antibody. The cells were then fixed, followed by incubation with anti-rabbit Alexa Fluor-568 antibody. The nuclei were stained with DAPI, and the cells were imaged (scale bar for zoomed image = 10 μm).

Table S1: Primers used in the site-directed mutagenesis.

Mutant	Primer	Nucleotide Sequence (5'-3')	Plasmid
P74A	Forward	GTGCAAGGACAAAAACCTA**GCC**GACTACAGCTGTAAGGTCTT	pcDNA5-E1-FLAG
	Reverse	TGTCCTGGTCTTCCTGCGCC**GGC**GGGCGGGTAGTCCATGTTAT	
F192A	Forward	ATAACATGGACTACCCGCCC**GCC**GGCGCAGGAAGACCAGGACA	pcDNA5-E1-FLAG
	Reverse	TGTCCTGGTCTTCCTGCGCC**GGC**GGGCGGGTAGTCCATGTTAT	
R206A	Forward	GGACAATTTGGCGATATCCAAAGT**GC**CACACCTGAGAGTAAAGACGTCTAT	pcDNA5-E1-FLAG
	Reverse	ATAGACGTCTTTACTCTCAGGTGTG**GC**ACTTTGGATATCGCCAAATTGTCC	
N216A	Forward	CCTGAGAGTAAAGACGTCTATGCT**GCC**ACACAACTGGTACTGCAGAGACCG	pcDNA5-E1-FLAG
	Reverse	CGGTCTCTGCAGTACCAGTTGTGT**GGC**AGCATAGACGTCTTTACTCTCAGG	
Q218A	Forward	GAGTAAAGACGTCTATGCTAATACA**GCC**CTGGTACTGCAGAGACCGGCTGC	pcDNA5-E1-FLAG
	Reverse	GCAGCCGGTCTCTGCAGTACCAG**GGC**TGTATTAGCATAGACGTCTTTACTC	
F192A-R206A	Forward	ATGGACTACCCGCCC**GC**TGGCGCAGGAAGACCAGGACAATTTGGCGATATCCAA AGT**GC**CACACCTGAGAGT	pcDNA5-E1-FLAG
	Reverse	ACTCTCAGGTGTG**GC**ACTTTGGATATCGCCAAATTGTCCTGGTCTTCCTGCGCCA**G** **C**GGGCGGGTAGTCCAT	
F192A-N216A	Forward	ATAACATGGACTACCCGCCC**GCC**GGCGCAGGAAGACCAGGACA	pcDNA5-E1-N216A-FLAG
	Reverse	TGTCCTGGTCTTCCTGCGCC**GGC**GGGCGGGTAGTCCATGTTAT	
L19A	Forward	GCTGCTGCTGCTCTTCCTGCTGCTG**GC**CTTCCTGCTGCCCACCCTGTGGTT	pcDNA5-AGPAT1-HA
	Reverse	AACCACAGGGTGGGCAGCAGGAAG**GC**CAGCAGCAGGAAGAGCAGCAGCAGC	
Y78A	Forward	CTAATGCTGCTCCACATCAAA**GC**CCTGTACGGGATCCGAGTGGAG	pcDNA5-AGPAT1-HA
	Reverse	CTCCACTCGGATCCCGTACAGG**GC**TTTGATGTGGAGCAGCATTAG	
L19A-Y78A	Forward	GCTGCTGCTGCTCTTCCTGCTGCTG**GC**CTTCCTGCTGCCCACCCTGTGGTT	pcDNA5-Y78A-AGPAT1-HA
	Reverse	AACCACAGGGTGGGCAGCAGGAAG**GC**CAGCAGCAGGAAGAGCAGCAGCAGC	

Table S2: Top poses obtained from protein-protein docking.

Models	Dock Score	Confidence score
Pose-1	-383.35	309
Pose-2	-379.93	310
Pose-3	-368.51	308
Pose-4	-357.78	290
Pose-5	-336.73	300

The dock score for the HDOCK poses and the confidence score for the AlphaFold 3 poses are presented and these were used as the filtering criteria for selecting the poses for further studies.

Table S3: The binding free energy of the HDOCK and AlphaFold 3 poses as the MM-GBSA scores (kcal/mol).

	HDOCK	AlphaFold 3
Pose-1	-152.3	-62.5
Pose-2	-148.9	-85.6
Pose-3	-122.0	-82.7
Pose-4	-117.0	-45.0
Pose-5	-87.4	-76.7
